# Filtering and inference for stochastic oscillators with distributed delays

**DOI:** 10.1093/bioinformatics/bty782

**Published:** 2018-09-08

**Authors:** Silvia Calderazzo, Marco Brancaccio, Bärbel Finkenstädt

**Affiliations:** 1 Department of Statistics, University of Warwick, Coventry, UK; 2 Division of Biostatistics, German Cancer Research Center, Heidelberg, Germany; 3 Division of Neurobiology, Medical Research Council Laboratory of Molecular Biology, Cambridge, UK

## Abstract

**Motivation:**

The time evolution of molecular species involved in biochemical reaction networks often arises from complex stochastic processes involving many species and reaction events. Inference for such systems is profoundly challenged by the relative sparseness of experimental data, as measurements are often limited to a small subset of the participating species measured at discrete time points. The need for model reduction can be realistically achieved for oscillatory dynamics resulting from negative translational and transcriptional feedback loops by the introduction of probabilistic time-delays. Although this approach yields a simplified model, inference is challenging and subject to ongoing research. The linear noise approximation (LNA) has recently been proposed to address such systems in stochastic form and will be exploited here.

**Results:**

We develop a novel filtering approach for the LNA in stochastic systems with distributed delays, which allows the parameter values and unobserved states of a stochastic negative feedback model to be inferred from univariate time-series data. The performance of the methods is tested for simulated data. Results are obtained for real data when the model is fitted to imaging data on *Cry*1, a key gene involved in the mammalian central circadian clock, observed via a luciferase reporter construct in a mouse suprachiasmatic nucleus.

**Availability and implementation:**

Programmes are written in MATLAB and Statistics Toolbox Release 2016 b, The MathWorks, Inc., Natick, Massachusetts, USA. Sample code and *Cry*1 data are available on GitHub https://github.com/scalderazzo/FLNADD.

**Supplementary information:**

Supplementary data are available at *Bioinformatics online*.

## 1 Introduction

The time evolution of molecular counts of chemical species in a reaction network is formally described by a Markov jump process (MJP). Interest usually lies in inferring the reaction rates and the unobserved molecule counts of the network’s species, given experimental data observed at discrete time-intervals for some or all of the reactants. Biochemical reaction networks are complex and often involve many reactions and chemical species. This is in stark contrast to the fact that only small subsets of species can be observed, albeit indirectly through measurement processes involving e.g. fluorescent reporter protein imaging. Hence, model reductions of the full reaction network towards less parameter-intensive approaches that can feasibly be estimated from the experimental data are of considerable importance.

The introduction of time-delays, in fixed or distributed form, can approximate the network’s species and reaction events which are not of primary interest and thus reduce model complexity (see e.g. Ananthasubramaniam *et al.*, 2014; Heron *et al.*, 2007; Korenčič *et al.*, 2012; Monk, 2003). Furthermore, it is well known that delays, as well as negative feedback, non-linearity and appropriate time-scales for the network’s reactions are necessary for the onset of oscillations in mathematical models (Cao *et al.*, 2016; Novák and Tyson, 2008). Delays can account for non-observable intermediate species or for time-intervals during which there are no measurements relating to the products of reactions. From the mathematical modelling point of view, this implies that the Markov property holds on a longer time-interval which extends up to an assumed maximum delay time.

A further main challenge in this field is due to the intractability of the transition densities of the MJP. Approximations in continuous state-space are available when suitable assumptions on the system size hold, and in particular, inferential applications have been considered for the chemical Langevin equation (CLE) (Golightly and Wilkinson, 2005, 2010; Heron *et al.*, 2007) and the linear noise approximation (LNA) (Fearnhead *et al.*, 2014; Finkenstädt *et al.*, 2013; Komorowski *et al.*, 2009; Stathopoulos and Girolami, 2013). For a recent detailed review of modelling and inferential methods for stochastic biochemical systems see Schnoerr *et al.* (2017). The CLE aims at matching the infinitesimal mean and variance of the original MJP, while the LNA performs a linearization which leads to tractable Gaussian transition densities. Fearnhead *et al.* (2014) find that in cases where the dynamics are non-linear, parameter inference via the LNA is improved by filtering, i.e. by replacing the mean and variance estimates of the process with their predicted value given the past observations.

The LNA for models with distributed delays has recently been derived by Brett and Galla (2013), but has not been suggested for filtering and inferential purposes, while it appears that a filtering methodology so far has only been suggested for fixed delays (Gopalakrishnan *et al.*, 2011). Here we develop a novel filtering algorithm that is based on the LNA and is generally applicable to stochastic systems comprising distributed delays, with a focus on dynamic state-space models for chemical reaction networks.

We first introduce biochemical reaction networks, their exact mathematical description and the CLE and LNA approximations. We then consider their CLE and LNA approximation in the broader framework of state-space models, and illustrate the LNA updating algorithm in the context of non-delayed systems. We present our novel extension, where the methodology is tested on simulated data and then applied to experimental data. Here we focus on providing an example of a stochastic transcriptional translational feedback loop (TTFL) to describe the expression dynamics of the circadian gene *Cry*1. The methodology is used to infer parameters of the TTFL of *Cry*1 from experimental time-series data observed in a mouse suprachiasmatic nucleus (SCN) tissue (Brancaccio *et al.*, 2013).

## 2 Materials and methods

### 2.1 Reaction networks and their approximations

Consider a reaction network defined by a set of chemical species participating in a set of chemical reactions. Let *p* and *r* denote the total number of species and reactions, respectively. The state of the process *X*(*t*) can be defined as (Anderson and Kurtz, 2011)

$$X(t)=X(0)+SU\left(\int_{0}^{t}h(X(s),c)ds\right),$$

where the vector of random variables $X(t)={\left(X_{1}(t),\ldots ,X_{p}(t)\right)}^{T}$ defines the number of molecules at time *t* of the species participating in the reaction network, U(·) is a vector of *r* independent inhomogeneous Poisson processes counting the occurrence of the *r* reactions and its argument defines its mean, *S* is the *p *×* r* stoichiometry matrix whose elements $s_{i,k}$ denote the difference in the number of molecules of the *i*-th species produced and consumed by the *k*-th reaction. The vector $h(X(s),c)={\left(h_{1}(X(s),c_{1}),\ldots ,h_{r}(X(s),c_{r})\right)}^{T}$ contains the reaction hazards where *c_k_* denotes the rate constant of the *k*-th reaction. The resulting process *X*(*t*) is a MJP in continuous time and discrete state-space, and its Kolmogorov’s forward equation, or chemical master equation (CME) (Gillespie, 1992), provides an exact description of the system. As it can only be explicitly solved in rare cases (see the review in McQuarrie, 1967), approximations help to overcome the need for highly computationally demanding inferential procedures. The CLE, or diffusion approximation, exploits the multivariate normal approximation of the vector of independent Poisson random variables *U*. The approximation holds under suitable assumptions concerning the number of reaction occurrences, and leads to the stochastic differential equation form (Anderson and Kurtz, 2011)

(1) $$dX(t)=g(X(t),c)dt+\sqrt{A(X(t),c)}dB(t),$$

where *dB*(*t*) is a *p*-dimensional Wiener process, and

g(X(t),c)=Sh(X(t),c)

$$A(X(t),c)=S\;\text{diag}\{h(X(t),c)\}S^{T}.$$

The drawback of the CLE approximation is that explicit solutions for the transition densities are again rare (see e.g. Wilkinson, 2012).

Gaussian transition densities can be obtained with the LNA (Kurtz, 1972; Van Kampen, 1992) which also exploits the approximate normality of the underlying Poisson process, but replaces the hazard function by its first order Taylor expansion about the deterministic limit of the process, thus effectively eliminating non-linearities. Assuming X(0)∼N(ρ(0),P(0)), where *ρ* denotes the deterministic limit, it can be shown that

$$X(t)\overset{\cdot }{∼}N(\rho (t),P(t))$$

such that the mean and the variance are the solutions of

(2) dρ(t)=g(ρ(t),c)dt

(3) $$dP(t)=J_{g}(\rho (t),c)P(t)dt+P{\left(t\right)}^{T}J_{g}{\left(\rho (t),c\right)}^{T}dt+A(\rho (t),c)dt,$$

where *J_g_* is the Jacobian of g(·) (see e.g. Anderson and Kurtz, 2011, for a rigorous derivation). The LNA matches exactly the first two moments provided by the CME for systems including reactions up to the first order, as well as for a subset of systems including second-order reactions (Grima, 2015). In the other cases, the LNA also approximates the mean and variance, as they depend on the higher moments (see e.g. Gillespie and Golightly, 2016; Grima, 2012, 2015).

### 2.2 Filtering and inference for the LNA

Inference from experimental data requires the formulation of a measurement equation. If light intensities generated by fluorescent reporter constructs are recorded, we may assume that observations are proportional to the unobserved molecular abundance and subject to additive Gaussian measurement error with unknown variance (see Finkenstädt *et al.*, 2013; Folia and Rattray, 2017; Hey *et al.*, 2015; Komorowski *et al.*, 2009)

(4) $$Y_{t}=FX_{t}+\epsilon_{t},$$

where $\epsilon_{t}∼MVN(0,\Sigma_{\epsilon })$, *F* is a *q *×* p* matrix (where *q* is the dimension of *Y* and *p* the dimension of *X*). The Markov structure of the process together with the measurement process in (4) formally leads to a state-space model, which provides a framework for inference. Let *ψ* denote the set of all parameters, namely the vector of reaction rates *c*, the variance matrix $\Sigma_{\epsilon }$ and the scale parameters in *F*. For random variables *X* and *Y*, let π(x) define the probability density function of *X*, and π(x|y) the conditional probability density function of *X* given *Y*. Furthermore, let $Y_{0:T}=Y_{0},Y_{\Delta_{t}},\ldots ,Y_{T}$ and $X_{0:T}=X_{0},X_{\delta_{t}},\ldots ,X_{T}$, where Δ_*t*_ is the time-interval of the observations, *δ_t_* some suitable discretization interval for the unobserved states dynamics and *T* is the total observation time. Inference typically involves estimating the model parameters *ψ* and the hidden states $X_{0:T}$. According to Bayes’ theorem, their joint posterior distribution is

$$\pi (\psi ,x_{0:T}|y_{0:T})=\frac{\pi (y_{0:T}|x_{0:T},\psi )\pi (x_{0:T}|\psi )\pi (\psi )}{\pi (y_{0:T})}.$$

If the focus of inference lies on estimating the model parameters, their marginal posterior distribution $\pi (\psi |y_{0:T})$ can be obtained by integrating out the hidden states, while inference on the hidden states can be performed by computing the smoothing density $\pi (x_{0:T}|y_{0:T},\psi )$ (see e.g. Doucet and Johansen, 2009; Wilkinson, 2012 and the Supplementary Section 1). In Gaussian dynamic linear models, all densities involved are Gaussian and the marginal likelihood $\pi (y_{0:T}|\psi )$ is available in a closed form provided by the well-known Kalman filtering methodology (Kalman, 1960). However, in reaction networks leading to the CLE representation of (1), Gaussianity is lost due to the dependence of the diffusion term A(·) on the system state. Furthermore, when the hazard function h(·) is non-linear, i.e. for reactions of order greater than one, only an approximate estimate of the mean and variance of the process is generally available.

In the filtering literature, non-linear and/or non-Gaussian systems have been approached with a variety of methods, e.g. the extended and second-order extended Kalman filter (see e.g. Jazwinski, 2007; Särkkä, 2013; Singer, 2002), the unscented Kalman filter (Julier and Uhlmann, 1997) and particle filters (see e.g. Andrieu *et al.*, 2010; Doucet and Johansen, 2009; Golightly and Wilkinson, 2011). Here we focus on the extended Kalman filter for time-continuous unobserved states, namely the extended Kalman–Bucy filter (EKBF) (see e.g. Kulikov and Kulikova, 2014; Singer, 2002 and references therein). Such choice can be motivated by its link to the LNA as follows.

Assume, for ease of notation, that the parameters *ψ* are set to some fixed value. In practice, parameter estimation can be performed via e.g. a Bayesian Markov chain Monte Carlo (MCMC) algorithm. The EKBF performs an update (restart) of the LNA mean and variance estimates at each observation time-point. Suppose $\rho (t)=E[X(t)|y_{0:t}]$ and $P(t)=\text{Var}[X(t)|y_{0:t}]$ in (2) and (3), respectively. Assuming that $\pi (x_{t}|y_{0:t})$ is approximately Gaussian, linearity of the LNA and of the measurement process implies that $\pi (x_{t+\Delta_{t}}|y_{0:t})$ and $\pi (y_{t+\Delta_{t}}|y_{0:t})$ are Gaussian, the latter with mean $F\rho_{t+\Delta_{t}}$, and variance $FP_{t+\Delta_{t}}F^{T}+\Sigma_{\epsilon }$. Estimates of the mean and variance of $\pi (x_{t+\Delta_{t}}|y_{0:t+\Delta_{t}})$ are obtained by a Kalman filtering step

(5) $$\rho_{t+\Delta_{t}}^{*}=\rho_{t+\Delta_{t}}+C(y_{t+\Delta_{t}}-F\rho_{t+\Delta_{t}})$$

(6) $$P_{t+\Delta_{t}}^{*}=P_{t+\Delta_{t}}-CFP_{t+\Delta_{t}},$$

where $C=P_{t+\Delta_{t}}F^{T}{\left(FP_{t+\Delta_{t}}F^{T}+\Sigma_{\epsilon }\right)}^{-1}$ is the adaptive coefficient. The current mean and variance estimates $\rho_{t+\Delta_{t}}$ and $P_{t+\Delta_{t}}$ are then replaced by their optimal estimates $\rho_{t+\Delta_{t}}^{*}$ and $P_{t+\Delta_{t}}^{*}$, and computations are iterated up to *T*. The update Equations (5) and (6) can be derived from approximate Gaussianity of the joint distribution $\pi (x_{t+\Delta_{t}},y_{t+\Delta_{t}}|y_{0:t})$, and the subsequent conditioning upon $Y_{t+\Delta_{t}}$. For further details see e.g. Jazwinski (2007), Särkkä (2013) and the Supplementary Section 2.

### 2.3 Extension to systems with distributed delays

Distributed delays can be introduced in the hidden state equation to account for a dependency on a (finite or infinite) collection of past states that are arbitrarily distant in time.

We focus on systems where a set of intermediate transformations of the species of interest can be well approximated by the Goodwin oscillator ordinary differential equations (ODEs) (Goodwin, 1965), which can be explicitly solved. The resulting system is a reduced reaction network, in which the hazard accounting for the transcriptional process receives as an input its past expression level, weighted according to the delay distribution. As a Hill function is assumed for transcription, this leads to a delay entering non-linearly in the transcriptional hazard. A formal derivation of the model is provided in the Supplementary Section 3. Further motivation or pursuing this modelling framework is provided in Section 3.1.

Brett and Galla (2013) derive a CLE and a (non-restarted) LNA for a modelling framework in which the delays are included linearly in the reaction hazards. Such scenario may arise under the assumption that the product of a reaction is not available for a non-negligible random time-interval, as well as under alternative modelling assumptions e.g. for an alternative target state in the Goodwin oscillator system.

To derive the LNA approach for our modelling scenario, we follow an approach analogous to Brett and Galla (2013), and divide the reactions into two groups: the set of *w* reactions with distributed delays, with stoichiometry *S_d_* of dimension *p *×* w* and hazard vector *h_d_* of length *w*, and the set of *z* reactions not involving delayed species, with stoichiometry matrix $S_{\bar{d}}$ of dimension *p *×* z*, and hazard vector $h_{\bar{d}}$ of length *z*. We have $S=[S_{d}|S_{\bar{d}}]$ and for the hazards

$$h_{d}\left(\int_{-\infty }^{t}X(s)\cdot K(t-s)ds\right)=\left[\begin{array}{c} h_{1}\left(\int_{-\infty }^{t}X(s)\cdot K(t-s)ds\right)\\ \vdots \\ h_{w}\left(\int_{-\infty }^{t}X(s)\cdot K(t-s)ds\right) \end{array}\right],$$

where $K(\cdot )={\left[K_{1}(\cdot ),\ldots ,K_{p}(\cdot )\right]}^{T}$ is a vector of delay densities, one for each *X_i_*. If *X_i_* is not delayed, *K_i_* has a point mass density at 0. The hazard vector of the non-delayed reactions is simply $h_{\bar{d}}(X(t))={\left[h_{w+1}(X(t)),\ldots ,h_{w+z}(X(t))\right]}^{T}$. We have dropped the dependence on the reaction rates *c* for ease of notation. It is practical to assume that the delay distribution is truncated at some maximum delay time *τ_m_*. Given the knowledge of the past states of the system, the hazards are deterministically defined and the CLE for the reaction network is

(7) $$\begin{array}{cc} dX(t)= & g(X(t))dt+f\left(\int_{t-\tau_{m}}^{t}X(s)\cdot K(t-s)ds\right)dt\\ & +\sqrt{l(X(t))+q\left(\int_{t-\tau_{m}}^{t}X(s)\cdot K(t-s)ds\right)}dB(t), \end{array}$$

where *dB*(*t*) is as usual a *p*-dimensional Wiener process, and

$$g(\cdot )=S_{\bar{d}}h_{\bar{d}}(X(t))$$

$$f(\cdot )=S_{d}h_{d}\left(\int_{t-\tau_{m}}^{t}X(s)\cdot K(t-s)ds\right)$$

$$l(\cdot )=S_{\bar{d}}\text{diag}[h_{\bar{d}}(X(t))]S_{\bar{d}}^{T}$$

$$q(\cdot )=S_{d}\text{diag}\left[h_{d}\left(\int_{t-\tau_{m}}^{t}X(s)\cdot K(t-s)ds\right)\right]S_{d}^{T}.$$

The full state-space model is hence given by the continuous approximation for the dynamics of the unobserved molecule counts in (7) together with the measurement Equation (4).

The derivation of the filtering algorithm poses two difficulties: (i) the linearization of the functions incorporating the delayed species, and (ii) the need to update, at each observation time *t*, all estimates in the past until time $t-\tau_{m}$. Problem (i) is addressed by a first order Taylor expansion of g(X(t)) and l(X(t)) about ρ(t), and of $f\left(\int_{t-\tau_{m}}^{t}X(s)\cdot K(t-s)ds\right)$ and $q\left(\int_{t-\tau_{m}}^{t}X(s)\cdot K(t-s)ds\right)$ about $\int_{t-\tau_{m}}^{t}\rho (s)\cdot K(t-s)ds$. The expansions are plugged into the mean and variance equations, thus allowing their propagation under linearity. A detailed derivation of the mean and variance equations is provided in the Supplementary Section 4. Optimal estimates of ρ(t) and *P*(*t*) for $t\in [0,\tau_{m}]$, required for the initialization of the algorithm, are generally not available in practice and require further modelling.

Problem (ii) can be approached by performing a Kalman update analogous to (5) and (6). Assuming approximate Gaussianity of the joint distribution $\pi (x_{t+\Delta_{t}-\tau_{m}:t+\Delta_{t}},y_{t+\Delta_{t}}|y_{0:t})$ and conditioning on $Y_{t+\Delta_{t}}$, implies that $\pi (x_{t+\Delta_{t}-\tau_{m}:t+\Delta_{t}}|y_{0:t+\Delta_{t}})$ is approximately Gaussian with mean $\rho_{t+\Delta_{t}-\tau_{m}:t+\Delta_{t}}^{*}$ and variance $P_{t+\Delta_{t}-\tau_{m}:t+\Delta_{t}}^{*}$, where

$$\rho_{t+\Delta_{t}-\tau_{m}:t+\Delta_{t}}^{*}=\rho_{t+\Delta_{t}-\tau_{m}:t+\Delta_{t}}+C(y_{t+\Delta_{t}}-F\rho_{t+\Delta_{t}})$$

$$P_{t+\Delta_{t}-\tau_{m}:t+\Delta_{t}}^{*}=P_{t+\Delta_{t}-\tau_{m}:t+\Delta_{t}}-CFP_{t+\Delta_{t},t+\Delta_{t}-\tau_{m}:t+\Delta_{t}}$$

with coefficient of adaptation *C*

$$C=P_{t+\Delta_{t}-\tau_{m}:t+\Delta_{t},t+\Delta_{t}}F^{T}{\left(FP_{t+\Delta_{t}}F^{T}+\Sigma_{\epsilon }\right)}^{-1}.$$

At $t+\Delta_{t}$ we therefore update, conditional on $y_{0:t+\Delta_{t}}$, the unobserved state mean and variance backwards until $t+\Delta_{t}-\tau_{m}$. At the end of the observation time *T* such updates effectively provide a ‘partial’ smoothing density, as the moments of $x_{T-\tau_{m}:T}$ are conditional on $y_{0:T}$, those of $x_{T-\tau_{m}-\Delta_{t}:T-\tau_{m}}$ are conditional on $y_{0:T-\Delta_{t}}$, etc.

The complexity introduced by the distributed delay comes at higher computational cost. When the dimension of *X* and, more crucially, the number of unobserved states included in the maximum delay time is large, the algorithm can be significantly slower than in the non-delayed case. This point is further investigated in Section 3.2.

## 3 Application

### 3.1 Model

We consider a stochastic transcriptional and translational feedback loop (TTFL) for a circadian clock that is represented by the self-inhibition of transcription after a Gamma distributed delay time *τ_p_*. The delay accounts for nuclear export, protein synthesis and nuclear import (see the Supplementary Section 3 for a formal derivation). We assume maximum delay time, $\tau_{m}=30$ h, such that possible dependence on past states is limited to just over a day, i.e. the cycle length of the system. Furthermore, we assume that a Hill function can approximate the relationship between the amount of available inhibitor and the transcriptional rate output, i.e.

$$\nu \left(\int_{t-\tau_{m}}^{t}X(s)\cdot K(t-s)ds\right)=\frac{R_{max}}{1+{\left(\frac{\int_{t-\tau_{m}}^{t}X(s)\cdot K(t-s)ds}{K_{pc}}\right)}^{n}}.$$

where *ν* is thus the transcription function. The parameter *R_max_* is the maximum achievable transcription rate, *n* is the Hill coefficient and is related to the number of binding sites present in the promoter region of the regulated gene and *K_pc_* is called the dissociation coefficient, or threshold, and represents the amount of input required to decrease the output of the transcriptional function by 50%. The Hill function can be formally derived under the assumption that binding and unbinding reactions of the inhibitor to the promoter happen at a fast time-scale, if compared to the time-scale of the transcriptional reaction (see e.g. Tkačik and Walczak, 2011).

The elimination of the intermediate species by means of the distributed delay and the use of a Hill type transcription function, leads to a reduced reaction network which consists of the following two reactions

(8) $$R_{1}:\emptyset^{\nu \left(\int_{t-\tau_{m}}^{t}X(s)\cdot K(t-s)ds\right)}\to X$$

(9) $$R_{2}:X\to \mu \emptyset ,$$

where *X* denotes the mRNA, K(·) is the truncated Gamma probability density function and *μ* is the mRNA degradation rate. Note that a key assumption has been made by considering the transcriptional process as the relevant source of intrinsic stochasticity.

The continuous state-space approximation of this model, combined with a measurement equation, is

$$Y_{t}=\kappa \int_{t-\Delta_{t}}^{t}X(s)ds+\epsilon_{t}$$

$$dX(t)=[\nu (\int_{t-\tau_{m}}^{t}X(s)\cdot K(t-s)ds)-\mu X(t)]dt+\sqrt{\nu (\int_{t-\tau_{m}}^{t}X(s)\cdot K(t-s)ds)+\mu X(t)}dB(t),$$

where $\epsilon_{t}∼N(0,\sigma_{\epsilon }^{2})$, and *κ* is the proportionality factor relating the unobserved molecular process to the light intensity. Here, the measurement Equation (4) has been modified to reflect a measurement process which involves integration of the light signal (see also Folia and Rattray, 2017). The model requires the specification of the initial condition, i.e. the mean and variance of $\pi (x_{0:\tau_{m}}|y_{0:\tau_{m}})$. To keep the dimensionality of the parameter space to a minimum, we assume a step function for the transcription rate *ν*, thus eliminating the dependence on past mRNA (Hey *et al.*, 2015; Jenkins *et al.*, 2013). Further details are provided in the Supplementary Section 5.

### 3.2 Simulation study

**Fig. 1.**
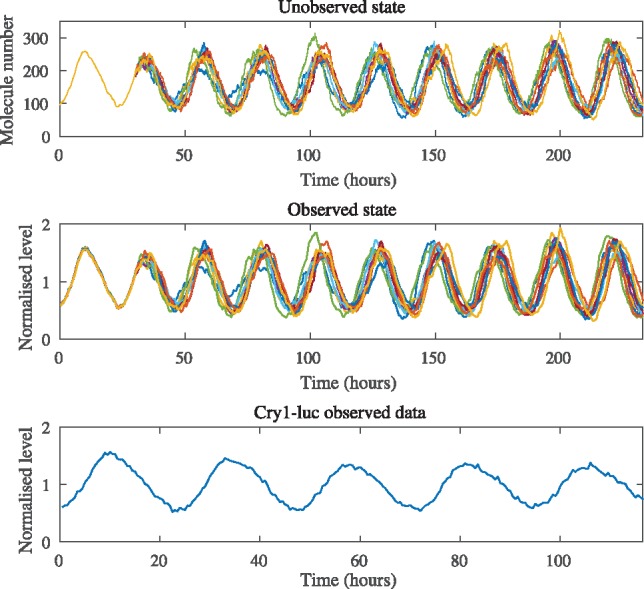
Top: SSA-type simulations for the reactions in (8) and (9). Center: molecule counts are rescaled by their mean level, integrated over 0.5 h and corrupted with measurement error. The assumed levels of signal to noise ratio is 100. Bottom: experimental *Cry1-luc* imaging time-series, aggregated, de-trended and normalized

We use the stochastic simulation algorithm (SSA) (Gillespie, 1977) to simulate approximately the dynamics of this reaction network where the hazard for the reaction in (8) is computed by evaluating the integral accounting for the delay up to the maximum delay time, each selected reaction is then assumed to take place immediately. As initial condition, we adopt the first 30 h of real observations for the *Cry1-luc* imaging data, which was rescaled, aggregated and de-trended. Values of mRNA are simulated and stored at fixed time-intervals of duration of length 0.01 h, and, to mimic the integration of camera light, are summed over 0.5 h, divided by their mean level, and corrupted with normal measurement error for two chosen levels of signal to noise ratio, i.e. 20 and 100. Figure 1 shows the simulated time-series for 10 replicates of the simulation algorithm for signal to noise ratio 100. Supplementary Figure S1 shows the simulated trajectories for signal to noise ratio 20.

#### 3.2.1 Filtering performance

We start by verifying the performance of the filtering methodology by setting the parameters to their true values. Computational practice requires some time-discretization of the ODEs involved. We adopt the Euler discretization method assuming *δ_t_* to control the step-size of the approximation. Supplementary Figure S2 provides a graphical illustration of the filter performance for $\delta_{t}=0.1$ on one sample simulated time-series. We note overall a good precision and, as expected, a decreased variability in the smoothing distribution. To compare the behaviour of the filter for different values of *δ_t_*, we compute the empirical coverage of both the predictive and the partial smoothing densities at level 95%. Results for the two levels of measurement error are provided in Supplementary Table S1. We observe an improved precision, i.e. a decreased discrepancy between nominal and empirical coverage, as *δ_t_* approaches its true value of 0.01 h, and particularly for the partial smoothing density under the lower measurement error scenario. The improvement is slower for values of *δ_t_* below 0.1 h, at which coverages for all cases are already between 94 and 96%. Furthermore, any improved coverage comes at higher computational cost. Supplementary Table S2 provides average running times of the filter. We observe that the rate of increase of the computational expense increases as *δ_t_* decreases, where the computation of the covariance of the unobserved states accounts for a significant proportion of the total running time. We note that alternative ODE solvers may be employed, and that the balance between precision and computational costs needs to be assessed in each case.

#### 3.2.2 Inference performance

We study the performance of the proposed filter for the purpose of Bayesian inference by designing a MCMC algorithm and applying it to the last five cycles of the simulated data (Fig. 1) with the aim of retrieving the values of the parameters used for the simulations. The MCMC scheme details are given in the Supplementary Section 6. Based on our results above and to balance the trade-off between realistic running times and filtering performance we choose to work with values $\delta_{t}\geq 0.1$ h. Visual investigation of the univariate log-likelihoods (see Supplementary Figs S3 and S4) suggests a minor effect of the choice of *δ_t_* on most of the parameters involved, except $\sigma_{\epsilon }$ and, more marginally, *κ*. This result motivates the design of a delayed acceptance MCMC algorithm (Christen and Fox, 2005; Golightly *et al.*, 2015), which allows to exploit the fast likelihood computation provided by the filter for $\delta_{t}=0.5$ h to explore the parameter space, but finally accepts values according to the likelihood provided by the filter for $\delta_{t}=0.1$ h.

For more efficient sampling we reparameterize the model by moving *κ* from the observation to the state equation. We generally adopt a $N(0,10^{2})$ prior density for all model parameters on the logarithmic scale, with the exceptions of the Hill coefficient, where we assume log(n) to have prior $N(\text{log}(1.5),5^{2})$ and the degradation rates during and after the initial condition, $\text{log}(\mu_{0})$ and log(μ), for which we specify a $N(\text{log}(0.58),0.5^{2})$ prior. The initial condition dispersion coefficient log(β), variance $\text{log}(\beta \text{Var}[X_{0}])$ and log(κ) are assumed to have a $N(0,20^{2})$ prior (see Supplementary Section 5 for further details on the initial condition model specification). An informative prior centred at the true simulation value and with unit SD is assumed for the measurement error SD, $\text{log}(\sigma_{\epsilon })$. Finally, the delay mean and SD are assigned *U* (0, 23) and *U* (0, 20) prior densities, respectively, both expressed in hours. This can be justified by assuming that the cellular product of the previous circadian cycle is feeding into the dynamics of the next cycle. Hence, most priors have been set to be diffuse within biologically realistic ranges. A certain amount of prior information seems to be required to robustify the inferential process. In particular, prior information on the reporter protein half-life is provided by Yamaguchi *et al.* (2003) although here we assume a larger SD to cope with the approximate nature of our model in particular as the reporter process is not explicitly modelled. An informative prior for the SD of the measurement error has been deduced by measurements from an additional light channel (M.Unosson, personal communication) where we adopt again a larger variance to indirectly account for processes not explicitly modelled. The initial conditions for the parameter chains are randomly drawn from the prior densities.

**Fig. 2.**
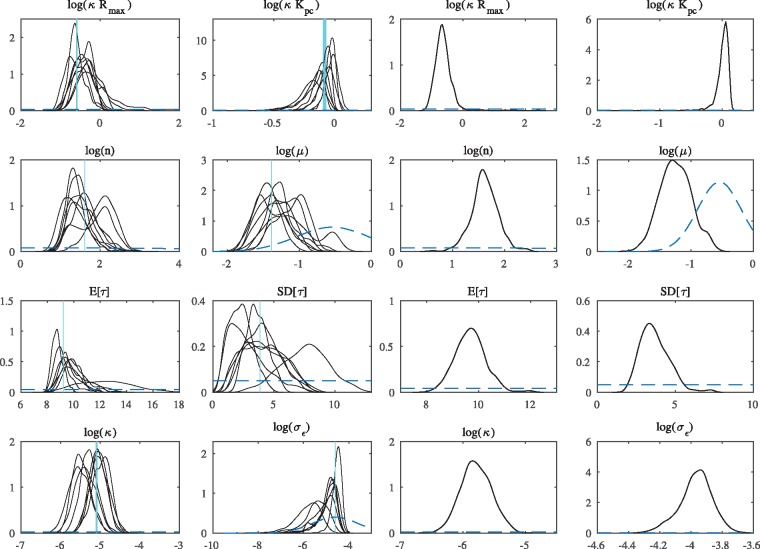
Left two columns: results for simulation study. Kernel densities’ estimates of the model parameters posterior densities, excluding the parameters of the initial condition. E[τ] and SD[τ] denote the mean and SD of the delay density *K*. The prior density is shown as a dashed line while the vertical line marks the true value. Results for the last five cycles of the simulated data shown in the central panel of Figure 1 (two chains are excluded due to non-convergence). Right two columns: results for observed *Cry1-luc* shown in bottom panel of Figure 1. Same notations and definitions as in panels on left

Figure 2 shows the estimated posterior densities of the model parameters for the simulated data seen in Figure 1. Two chains have been excluded due to lack of convergence, but inference seems overall satisfactory for the remaining chains. The results for the signal to noise ratio 20 scenario are provided in Supplementary Figure S5, and lead to analogous conclusions.

We have also investigated filter coverage and inference performance for increasing degrees of non-linearity by varying the Hill coefficient and setting it =7, 9, 11, and found that the estimation results are overall consistent with the results shown for *n *=* *5 (see Supplementary Tables S3–S5 and Figs S6–S11). We postulate that this is due to the use of the filtering step in the LNA, which seems to effectively counteract the effect of the increasing error in the LNA prediction that can be expected for higher degrees of non-linearity.

### 3.3 Inference for the circadian feedback loop in *Cry*1

The available data are time-series for the circadian gene *Cry*1, observed by mean of a transcriptional luciferase reporter construct *Cry1-luc* in a mouse SCN tissue over five days (Brancaccio *et al.*, 2013) (see bottom panel of Fig. 1). Light intensities are recorded by EM-CCD Camera (Hamamatsu) with an exposure time of 0.5 h, i.e. the image is the result of the photons hitting the camera in 0.5 h intervals (see Brancaccio *et al.*, 2013). A mean trend due to consumption of the luciferin substrate is accounted for by dividing the observations by a time-varying proportionality factor which assumes a linear decrease of ∼30% over five days. We apply the proposed model to a sample location in the SCN where the data are aggregated over a 2 × 2 pixel box which is broadly comparable to the size of a cell. Figure 2 provides the estimated posterior densities of the model parameters for the experimental data. Their interpretation has to be considered in the light of the fact that we have represented a complex genetic network of the circadian clock, which consist of several interwoven TTFs involving about 15 clock genes (Dibner and Schibler, 2015) by a delayed feedback loop fitted to imaging data of a single gene involved. The main achievement here over previous work is that we have provided the inferential methodology to identify such a model from time-series imaging data which can potentially serve as a realistic surrogate model to be fitted in a longitudinal or spatial fashion to quantify and compare intrinsic noise and to study between-cell variability. Of particular interest are the parameters associated with the delay distribution, where the mean influences the period of the oscillations, and the variance tunes the temporal precision of the clock period. The mean delay time here is estimated to be around 9.67 h [95% HPDI (8.46, 10.80)] which, although not statistically significant, is not far away from Korenčič *et al.* (2012) who find that a value of 8.25 h achieves circadian periodicity in a deterministic feedback loop model with discrete delay for *Per*2 self-inhibition. Furthermore, we estimate a value of around 3.56 h [95% HPDI (2.01, 5.39)] for the SD of the delay distribution. We note that the inferred posterior distribution of the parameter is concentrated away from 0, which would correspond to a fixed delay, suggesting that the assumption of a distributed delay is indeed tenable. We also hypothesize that this estimate may be relatively small as the experimental data are from the SCN, which is known to be the main pacemaker of the mammalian clock, but may be spatially varying over the SCN. Such further questions can be addressed on the basis of the TTFL modelling and inference approach proposed here.

The model fit is checked through inspection of the data posterior predictive distribution and the standardized residuals, where we obtain samples using a thinned set of parameter samples from the MCMC algorithm. Results are displayed in Supplementary Figure S12. The normal *q*–*q* plot reveals a slightly heavy upper tail, although this result does not seem of major concern nor to be due to a systematic under-performance on the filter, as also investigated in further yet unpublished work. Visual investigation and model diagnostics reveal no residual 24-h periodicity in the residuals after the first 30 h of initial condition (which we recall is only instrumental into obtaining a first estimate of the mean and covariance matrix), which indicates that the circadian oscillation is well explained by the model. However, a non-negligible residual periodicity of around 12 h can still be found. As pointed out to us by an anonymous reviewer, one possible explanation is that the model may not be capturing part of the non-linearity observed in the data. This may be caused by processes which are not explicitly included in the model such as the influence of inter-cellular signalling. The latter is relevant for *Per* transcription, and therefore for PER protein, which forms the PER/CRY complex effectively repressing *Cry*1 transcription. The transcription function assumed above can only account for repression by means of a single transcription factor. It may also be of relevance that Zhu *et al.* (2017) recently found a cell-autonomous 12 h clock in mammals.

## 4 Discussion

In this work we have addressed filtering and inference for state-space models with distributed delays, with a special focus on models arising from stochastic biochemical networks with stochastic oscillatory behaviour. The methodology is derived with focus on a stochastic self-inhibitory feedback loop with distributed delay, noting that this kind of model is known to achieve a substantial model reduction of complex gene networks and is of particular interest for modelling oscillatory molecular clocks. As such complex networks are never fully observed, model reduction is also important to facilitate inference from experimental data. The introduction of a distributed delay, as compared to a fixed delay, is beneficial as it provides a more realistic description of the process. The type of oscillatory processes here considered can indeed possess a certain degree of variability in their period that a fixed delay would not be able to characterize. We have extended the LNA and EKBF to provide a methodology which allows for sequential computation of the likelihood in such models. The resulting likelihood has a closed form and can be incorporated in a Bayesian MCMC algorithm for parameter inference. The performance of the methodology is tested on simulated data and first real results are shown here for an experimental *Cry1-luc* time-series from a mouse SCN (Brancaccio *et al.*, 2013).

The closest approach to date is perhaps the work of Heron *et al.* (2007), which performs inference on a closely related dynamical model for gene expression, where inference is based on the CLE description of the process. In contrast to our methodology, their approach does not allow to obtain a closed-form likelihood and requires sampling the parameters conditional on a sampled path of the unobserved states in the MCMC algorithm and vice-versa, rendering the inference problem very high-dimensional. As the parameters and unobserved states trajectories tend to be strongly correlated, sampling is likely to be less efficient (Golightly and Wilkinson, 2011).

It should be noted that moment-closure approximations (MA) represent an inferential approach of comparable computational cost which can also be of interest for our scenario. MA provide ODEs for the time evolution of the approximate moments of the process by truncation of higher order moments, and have been applied for inferential purposes in e.g. Zechner *et al.* (2012) and Fröhlich *et al.* (2016). The method can lead to physically implausible results, such as negative mean and variances, but Schnoerr *et al.* (2017) have recently shown an improved precision over the LNA for some simulation scenarios.

Alternative specifications for the transcription function can be investigated, e.g. Kim *et al.* (2014) assume that the inhibitory processes arise as a consequence of sequestration of the activator by the inhibitor. To date, modelling of the TTFL of circadian genes in the mammalian clock is either based on deterministic approaches, possibly comprising a simplification using a delay, or on stochastic descriptions which have not yet attempted statistical parameter inference from experimental time-series data (see Ananthasubramaniam *et al.*, 2014; DeWoskin *et al.*, 2015; Gonze *et al.*, 2005; Kim and Forger, 2012; Korenčič *et al.*, 2012; Relógio *et al.*, 2011). Our approach extends over existing mathematical modelling in that it provides a novel stochastic inferential approach for such system.

We note that our approach does not specifically take into account the fact that we observe reporter protein rather than the protein of interest, which is possibly a strong but necessary simplification. Inference is challenging due to the strong parameter correlation structure, where we hope to show in future work that joint estimation of the parameters for data at many more spatial locations across the SCN may substantially aid the inferential process.

The ability of the proposed methodology to address intrinsic stochasticity is of particular importance in models of biochemical oscillators. Indeed, the idea that noisy systems are more easily entrained to an external input has been investigated both theoretically (Steuer *et al.*, 2003) and experimentally (Ko *et al.*, 2010). The latter have studied the role of intrinsic noise in the SCN, and provided experimental evidence from a *Bmal*1-null mutant mouse that noise and extracellular signalling are sufficient to produce oscillations when the TTFL is disrupted. Further insight into the stochastic biochemical oscillators and their synchronization may be achieved by performing inference on the spatial distribution of the model parameters over the SCN. The methodology presented here will form the basis to be able to perform such an analysis.

## Supplementary Material

Supplementary Data
