# Supplementary material for: Filtering and inference for stochastic oscillators with distributed delays
Source: Bioinformatics. 2018 Sep 8;35(8):1380–7. doi: 10.1093/bioinformatics/bty782 (PMC6477979; doi:10.1093/bioinformatics/bty782)
Supplement: Supplementary Data [file bty782_supplementary_data.pdf]

Supplementary Information

Filtering and Inference for stochastic oscillators with distributed delays

S Calderazzo, M Brancaccio and B Finkenstädt

---

1. Filtering and smoothing densities for state-space models

In a state-space model the joint posterior distribution for the parameters and the hidden states, using Bayes' theorem, is given by

$$\pi(\psi, x_{0:T}|y_{0:T}) = \frac{\pi(y_{0:T}|x_{0:T}, \psi)\pi(x_{0:T}|\psi)\pi(\psi)}{\pi(y_{0:T})}.$$

If the focus of inference lies on estimating the model parameters, their marginal posterior distribution can be obtained by integrating out the hidden states. The properties of state-space models allow to write (see e.g. Doucet and Johansen, 2009; Wilkinson, 2012)

$$\begin{aligned}\pi(\psi|y_{0:T}) &\propto \pi(\psi)\pi(y_{0:T}|\psi)\\ &= \pi(\psi)\pi(y_0|\psi)\prod_{i=1}^{T/\Delta_t}\pi(y_{i\Delta_t}|y_{0:(i-1)\Delta_t}, \psi),\end{aligned}$$

where

$$\pi(y_0|\psi) = \int \pi(y_0|x_0, \psi)\pi(x_0|\psi)dx_0, \tag{S1}$$

and

$$\pi(y_{i\Delta_t}|y_{0:(i-1)\Delta_t}, \psi) = \int \pi(y_{i\Delta_t}|x_{i\Delta_t}, \psi)\pi(x_{i\Delta_t}|x_{(i-1)\Delta_t}, \psi)\pi(x_{(i-1)\Delta_t}|y_{0:(i-1)\Delta_t}, \psi)dx_{(i-1)\Delta_t:i\Delta_t}. \tag{S2}$$

The density $\pi(y_{0:T}|\psi)$ is the model likelihood, and is available in closed form when all the densities involved are Gaussian. In such case, indeed, the integrals (S1) and (S2) can be explicitly solved, resulting in turn in Gaussian densities. The mean and variance of these distributions are provided by Kalman filter recursions.

On the other hand, inference on the hidden states can be performed by obtaining the smoothing density of the model, i.e.

$$\pi(x_{0:T}|y_{0:T}, \psi) = \pi(x_T|y_{0:T}, \psi)\prod_{i=0}^{(T-\Delta_t)/\Delta_t}\pi(x_{i\Delta_t}|x_{i\Delta_t+\Delta_t}, y_{0:T}, \psi),$$

where again the properties of state-space models are exploited to write the density in a sequential form.

2. Derivation of the unobserved states approximate mean and variance equations in non-delayed systems

The mean and variance equations (2) and (3) in the main text can alternatively be derived from the Euler-Maruyama approximation over a time-interval δ_t of the unobserved states SDE (see Singer, 2006; Särkkä, 2007), i.e. the CLE (1) in the main text can be approximated as

$$X_{t+\delta_t} = X_t + g(X_t)\delta_t + a(X_t)\Delta B_t + o(\delta_t),$$

where $\Delta B_t = \sqrt{\delta_t} Z_t$, and Z_t is a standard normal. Letting $A(\cdot) = a(\cdot)a(\cdot)^T$ and dropping the terms of order $o(\delta_t^2)$, it follows that (Singer, 2006)

$$\text{(S3)} \qquad \mathrm{E}[X_{t+\delta_t}|y_{0:t}] \approx \mathrm{E}[X_t|y_{0:t}] + \mathrm{E}[g(X_t)|y_{0:t}]\delta_t$$

$$\text{(S4)} \qquad \mathrm{Var}[X_{t+\delta_t}|y_{0:t}] \approx \mathrm{Var}[X_t|y_{0:t}] + \mathrm{Cov}[X_t, g(X_t)|y_{0:t}]\delta_t + \mathrm{Cov}[g(X_t), X_t|y_{0:t}]\delta_t + \mathrm{E}[A(X_t)|y_{0:t}]\delta_t.$$

Let $\rho_t = \mathrm{E}[X_t|y_{0:t}]$, the first order Taylor expansion of $g(\cdot)$ and $A(\cdot)$ about ρ_t is

$$\text{(S5)} \qquad g(X_t) \approx g(\rho_t) + J_g(\rho_t)(X_t - \rho_t)$$

$$\text{(S6)} \qquad A(X_t) \approx A(\rho_t) + J_A(\rho_t)(X_t - \rho_t).$$

where J denotes the Jacobian matrix. The expansions (S5) and (S6), truncated at the first order, are then plugged into (S3) and (S4), allowing their propagation under linearity (Singer, 2006). Defining $P_t = \mathrm{Var}[X_t|y_{0:t}]$ and rearranging, the mean and variance equations read

$$\text{(S7)} \qquad \rho_{t+\delta_t} - \rho_t \approx g(\rho_t)\delta_t$$

$$\text{(S8)} \qquad P_{t+\delta_t} - P_t \approx J_g(\rho_t)P_t\delta_t + P_t^T J_g(\rho_t)^T\delta_t + A(\rho_t)\delta_t.$$

By dividing both sides of (S7) and (S8) by δ_t, and letting $\delta_t \to 0$, the time-continuous form of (2) and (3) in the main text is recovered.

3. Model derivation

Assume that the full system, accounting for transcription, nuclear export, translation, complex formation and nuclear import, can be described by the following set of ODEs, also known as the Goodwin oscillator (Goodwin, 1965),

$$\text{(S9)} \qquad dx_{M_g}(t) = \left[\nu(x_{P_p}(t)) - \mu x_{M_g}(t)\right] dt$$

$$\text{(S10)} \qquad dx_{P_1}(t) = \alpha[x_{M_g}(t) - x_{P_1}(t)]dt$$

$$\vdots$$

$$\text{(S11)} \qquad dx_{P_p}(t) = \alpha[x_{P_{p-1}}(t) - x_{P_p}(t)]dt,$$

where x_{M_g} denotes the mRNA counts, and $x_{P_1}, \ldots, x_{P_p}$ the counts of the intermediate species. Moreover, let $\nu(\cdot)$ denote the assumed transcription

function. It is shown in Smith (2010) that the system in (S9)-(S11), with initial condition

$$x_{M_g}(0) = \phi(0)$$
$$P_j(0) = \int_0^{\infty} \phi(-s) K_{j,\alpha}(s) ds, \quad j = 1, ..., p$$

where $\phi : (-\infty, 0] \to \mathbb{R}$ is bounded and continuous, is equivalent to

$$dx_{M_g}(t) = \left[\nu \left(\int_0^{\infty} x_{M_g}(t-s) K_{p,\alpha}(s) ds \right) - \mu x_{M_g}(t) \right] dt, \tag{S12}$$

with initial condition $x_{M_g}(\theta) = \phi(\theta)$, for $\theta \leq 0$, and

$$K_{p,\alpha}(s) = \frac{\alpha^p s^{(p-1)} e^{-\alpha s}}{(p-1)!}, \tag{S13}$$

is the probability density function of a $Ga(p, \alpha)$, evaluated at s. We partially follow El Cheikh *et al.* (2012) for the proof.
The intermediate species $x_{P_1}, ..., x_{P_p}$ ODEs can be explicitly solved. Indeed, the solution for $x_{P_1}(t)$ is given by

$$x_{P_1}(t) = \alpha e^{-\alpha t} x_{P_1}(0) + \int_{-\infty}^{t} \alpha e^{\alpha(t-u)} x_{M_g}(u) du.$$

As $t \to \infty$, the contribution of the initial condition tends to 0, and the remaining integral can be seen as a convolution between x_{M_g} and a $Ga(1, \alpha)$. Consider then an arbitrary x_{P_j}, for $j \in \{2, ..., p\}$, and suppose that $x_{P_{j-1}}(t)$ is the convolution between x_{M_g} and a $Ga(j-1, \alpha)$. The solution for $x_{P_j}(t)$ is equivalently given by

$$x_{P_j}(t) = \alpha e^{-\alpha t} x_{P_j}(0) + \int_{-\infty}^{t} \alpha e^{\alpha(t-u)} x_{P_{j-1}}(u) du,$$

where again, neglecting the initial condition, a convolution between $x_{P_{j-1}}$ and a $Ga(1, \alpha)$ is obtained. By induction, the additive property of the convolution, and the fact that the convolution of p independent $Ga(1, \alpha)$ is a $Ga(p, \alpha)$, it can be concluded that, as $t \to \infty$,

$$x_{P_p}(t) = (x_{M_g} * K_{p,\alpha})(t),$$

where K is defined as in (S13). By plugging the result into the mRNA equation, (S12) is obtained.

Two main assumptions are required for the previous result: first, all the translation and degradation rates of the intermediate states are assumed to be equal, and second, it is based on deterministic dynamics. If the degradation rates are different, we have a convolution of gamma densities having different rate parameters. We assume that this distribution can still however be reasonably approximated by a single Gamma density.

As for the deterministic form, we postulate that most of the stochasticity is generated by the mRNA state, given that cellular protein levels are generally much higher than mRNA counts (see e.g. Suter *et al.*, 2011), and replace the ODE for x_{M_g} with the stochastic MJP for X_{M_g}. The resulting stochastic model for the mRNA is an immigration and death process, whose macroscopic rate equation is given by (S12). The diffusion approximation

of such model for the mRNA and for an additional intermediate state, is employed for inferential purposes also in Heron *et al.* (2007).

4. Derivation of the unobserved states approximate mean and variance equations in systems incorporating delays

The derivation of the mean and variance equations for systems incorporating time-delayed species follows steps analogous to Section 2 for non-delayed systems. In particular, from an Euler-Maruyama approximation of the CLE (7) in the main text, after dropping the terms of order $o(\delta_t^2)$ we have that

$$\text{(S14)}\quad \begin{aligned} \mathrm{E}\left[X_{t+\delta_t}|y_{0:t}\right] &= \mathrm{E}\left[X_t|y_{0:t}\right] + \mathrm{E}\left[g(X_t)|y_{0:t}\right]\delta_t \\ &+ \delta_t \mathrm{E}\left[f\left(\sum_{s=(t-\tau_m+\delta_t)/\delta_t}^{t/\delta_t} X_{s\delta_t}\cdot K_{t-s\delta_t+\delta_t/2}\right)|y_{0:t}\right] \end{aligned}$$

$$\text{(S15)}\quad \begin{aligned} \mathrm{Var}[X_{t+\delta_t}|y_{0:t}] &= \mathrm{Var}[X_t|y_{0:t}] + \mathrm{Cov}\left[x_t, g(X_t)|y_{0:t}\right]\delta_t \\ &+ \mathrm{Cov}\left[g(X_t), X_t|y_{0:t}\right]\delta_t \\ &+ \mathrm{Cov}\left[X_t, f\left(\sum_s X_{s\delta_t}\cdot K_{t-s\delta_t+\delta_t/2}\right)|y_{0:t}\right]\delta_t \\ &+ \mathrm{Cov}\left[f\left(\sum_s X_{s\delta_t}\cdot K_{t-s\delta_t+\delta_t/2}\right), X_t|y_{0:t}\right]\delta_t \\ &+ \mathrm{E}\left[A\left(X_t, \sum_s X_{s\delta_t}\cdot K_{t-s\delta_t+\delta_t/2}\right)|y_{0:t}\right]\delta_t, \end{aligned}$$

where $K_{t-s\delta_t+\delta_t/2}$ is the discretised delay density evaluated at $t - s\delta_t + \delta_t/2$ and normalised, and

$$A\left(X_t, \sum_s X_{s\delta_t}\cdot K_{t-s\delta_t+\delta_t/2}\right) = l(X_t) + q\left(\sum_s X_{s\delta_t}\cdot K_{t-s\delta_t+\delta_t/2}\right).$$

Taylor-expand the nonlinear function $g(\cdot)$ about $\rho_t = \mathrm{E}[X_t|y_{0:t}]$, and $f(\cdot)$ about $\bar{\rho}_s = \sum_s \rho_{s\delta_t}\cdot K_{t-s\delta_t+\delta_t/2}$, up to the first order

$$g(X_t) \approx g(\rho_t) + J_g(\rho_t)(X_t - \rho_t)$$

$$\begin{aligned} f\left(\sum_s X_{s\delta_t}\cdot K_{t-s\delta_t+\delta_t/2}\right) &\approx f(\bar{\rho}_s) + J_f(\bar{\rho}_s) \\ &+ \sum_s (X_{s\delta_t} - \rho_{s\delta_t})\cdot K_{t-s\delta_t+\delta_t/2}. \end{aligned}$$

Performing analogous expansions for $l(\cdot)$ and $q(\cdot)$ about ρ_t and $\bar{\rho}_s$, respectively, it can be shown that

$$\mathrm{E}\left[A\left(X_t, \sum_s X_{s\delta_t}\cdot K_{t-s\delta_t+\delta_t/2}\right)|y_{0:t}\right] = A(\rho_t, \bar{\rho}_s).$$

Plugging the results into (S14) and (S15), we obtain

$$\begin{aligned}
\text{(S16)}\qquad \mathrm{E}[X_{t+\delta_t}|y_{0:t}] &\approx \mathrm{E}[X_t|y_{0:t}] \\
&\quad + \delta_t g(\rho_t) + J_g(\rho_t)\mathrm{E}[X_t - \rho_t|y_{0:t}]\delta_t + f\left(\bar{\rho}_s\right)\delta_t \\
&\quad + J_f\left(\bar{\rho}_s\right)\sum_s \mathrm{E}\left[(X_{s\delta_t} - \rho_{s\delta_t})|y_{0:t}\right]\cdot K_{t-s\delta_t+\delta_t/2}\delta_t \\
&= \rho_t + \delta_t g(\rho_t) + f\left(\bar{\rho}_s\right)\delta_t
\end{aligned}$$

$$\begin{aligned}
\text{(S17)}\qquad \mathrm{Var}[X_{t+\delta_t}|y_{0:t}] &\approx P_t + \delta_t\left[J_g(\rho_t)P_t + P_t^T J_g(\rho_t)^T\right] \\
&\quad + \delta_t\left[J_f\left(\bar{\rho}_s\right)\left(\sum_s P_{s\delta_t,t}\cdot K_{t-s\delta_t+\delta_t/2}\right)\right] \\
&\quad + \delta_t\left[\left(\sum_s P_{t,s\delta_t}\cdot K_{t-s\delta_t+\delta_t/2}\right)J_f\left(\bar{\rho}_s\right)^T\right] \\
&\quad + \delta_t A\left(\rho_t,\bar{\rho}_s\right).
\end{aligned}$$

The mean and variance prediction can be iterated until the next observation time-point, to obtain $\rho_{t:t+\Delta_t}$ and $P_{t:t+\Delta_t}$.

A time-continuous form for the evolution of the mean and variance can be derived by letting $\delta_t \to 0$. In particular, from (S16) and (S17), we have

$$\begin{aligned}
\frac{\rho_{t+\delta_t} - \rho_t}{\delta_t} &\approx g(\rho_t) + f\left(\bar{\rho}_s\right) \\
\frac{P_{t+\delta_t} - P_t}{\delta_t} &\approx \left[J_g(\rho_t)P_t + P_t^T J_g(\rho_t)^T\right] \\
&\quad + \left[J_f\left(\bar{\rho}_s\right)\left(\sum_s P_{s\delta_t,t}\cdot K_{t-s\delta_t+\delta_t/2}\right)\right] \\
&\quad + \left[\left(\sum_s P_{t,s\delta_t}\cdot K_{t-s\delta_t+\delta_t/2}\right)J_f\left(\bar{\rho}_s\right)^T\right] \\
&\quad + A\left(\rho_t,\bar{\rho}_s\right),
\end{aligned}$$

and, as $\delta_t \to 0$,

$$\begin{aligned}
\frac{d\rho(t)}{dt} &\approx g(\rho(t)) + f\left(\int_{t-\tau_m}^t \rho(s)\cdot K(t-s)ds\right) \\
\frac{dP(t)}{dt} &\approx \left[J_g(\rho(t))P(t) + P(t)^T J_g(\rho(t))^T\right] \\
&\quad + \left[J_f\left(\int_{t-\tau_m}^t \rho(s)\cdot K(t-s)ds\right)\left(\int_{t-\tau_m}^t P(s,t)\cdot K(t-s)ds\right)\right] \\
&\quad + \left[\left(\int_{t-\tau_m}^t P(t,s)\cdot K(t-s)ds\right)J_f\left(\int_{t-\tau_m}^t \rho(s)\cdot K(t-s)ds\right)^T\right] \\
&\quad + A\left(\rho(t),\int_{t-\tau_m}^t \rho(s)\cdot K(t-s)ds\right).
\end{aligned}$$

5. Initial condition

The observations obtained from time 0 to time τ_m require modelling which does not rely on past observations. The model assumed for the initial condition should be as parsimonious as possible, as the estimates obtained are only instrumental at obtaining a sensible initial estimate of the mean and variance of the system.

In our example, we assume a single change in transcription rate occurring around the observed peak time, thus eliminating the dependence on past mRNA. Formally, the unobserved state equation has the form

$$\begin{aligned} dX(t) = &\left[\mathrm{I}_{\{0:t_c\}}\nu_1 + \mathrm{I}_{\{t_c:\tau_m\}}\nu_2 - \mu_0 X(t)\right] dt \\ &+ \sqrt{\beta}\sqrt{\mathrm{I}_{\{0:t_c\}}\nu_1 + \mathrm{I}_{\{t_c:\tau_m\}}\nu_2 + \mu_0 X(t)}dB(t), \end{aligned}$$

where I is the indicator function, t_c is the time at which transcription switches from rate ν_1 to rate ν_2. Finally, β and μ_0 are the scale and the degradation rate for the initial condition, additionally introduced in order to avoid a possible source of bias in the estimation process due to model mismatch. Note that the parameter β plays the same role as the scale parameter κ in the main text (to see this, move κ from the observed state to the unobserved scale equation, and rescale the transcription function parameters accordingly), and thus we additionally drop κ from the observed state equation. The non-delayed system is treated as outlined in Section 2.2 of the main text.

6. MCMC details

The MCMC algorithm adopted has a pilot run of 3×10^4 iterations. For the first 10^3 iterations the transcription function parameters are updated independently with a random walk Metropolis-Hastings scheme, with a fixed variance for the gaussian proposals equal to 1. The remaining parameters are updated in two blocks, one for the initial condition parameters and one for the measurement parameters, each using a fixed diagonal covariance matrix for the proposals with diagonal entries equal to $1/d_k$, where d_k is the dimension of block $k = 1, 2$. From iteration 10^3, the transcription function parameters are sampled in an additional block. With probability 5%, the covariance matrix of each block is equal to $0.1^2/d_k$, while with probability 95%, it is equal to the covariance matrix of the previously accepted values times $1/d$ for the transcription function parameters block, and $2.38^2/d_k$ for the remaining blocks (see Roberts and Rosenthal, 2009). The unobserved states discretisation time-interval here is set to $\delta_t = 0.5$ h, to obtain a fast first exploration of the posterior density.

From iteration 3×10^4, a delayed acceptance component is additionally introduced (Christen and Fox, 2005; Golightly *et al.*, 2015; Sherlock *et al.*, 2017). In a delayed acceptance scheme, samples are first proposed according to a 'fast' likelihood evaluation, we choose in our case to adopt $\delta_t = 0.5$ h, and accepted or rejected according to the usual acceptance ratio of a Metropolis-Hastings scheme. If the proposed samples are accepted, a slower and more precise evaluation of the likelihood is performed, in our case δ_t is set to 0.1 h. The acceptance ratio of the nested step is, then, (Christen and

Fox, 2005; Golightly *et al.*, 2015)

$$\alpha_{DA} = \min\left\{1, \frac{\pi_{0.1}(y_{0:T}|\Psi^*)}{\pi_{0.1}(y_{0:T}|\Psi)}\frac{\pi_{0.5}(y_{0:T}|\Psi)}{\pi_{0.5}(y_{0:T}|\Psi^*)}\right\},$$

where Ψ^* is the proposed sample of parameters, accepted in the fast likelihood evaluation step, and Ψ the accepted sample from the previous iteration of the MCMC algorithm. Moreover, we denote by $\pi_{0.1}$ the likelihood under $\delta_t = 0.1$ h, and by $\pi_{0.5}$ the likelihood under $\delta_t = 0.5$ h. The choice of a delayed acceptance scheme is motivated by the fact that the proposed filter becomes computationally time-demanding as the chosen δ_t decreases. Indeed, we have a computational cost which is approximately 3.5 times higher under the $\delta_t = 0.1$ h case than in the $\delta_t = 0.5$ h one (see Table S2). Moreover, the profile likelihoods and filter performance seem to be already relatively satisfactory when $\delta_t = 0.5$, suggesting that proposals accepted at the first stage are likely to be accepted also at the nested stage, and thus the algorithm can be efficient for this application. Sherlock *et al.* (2017) also investigates efficiency of the delayed acceptance algorithm with respect to the jump size of the proposals. These can be can be larger than in a non-delayed scheme, as the acceptance/reject step of the first stage allows a faster exploration of the posterior density. In practice, we have found that the traditional multiplying constant of $2.38^2/d_k$ for the proposals covariance is already too large for the transcription function parameters block, and we have thus reduced it to $1/d_k$. This value has been kept constant through the pilot and the final run. In the pilot run, it seems to provide a satisfactory behaviour in exploration of the posterior density, and, for the later samples, we believe that the small change in the shape of target likelihood, as well as a relatively low observed acceptance rate, may motivate to not increase such value.

After the pilot run, we discard 10^5 iterations as a burn-in, and thin the chains by retaining one sample every 100 iterations.

We note that the evaluation of the likelihood may encounter occasional numerical problems, especially in the initial iterations, and for very low or high proposed values of the degradation rate. In such cases, the proposed values are rejected. We also note that mixing of the chains may be challenging for the transcription function parameters, as also suggested by the posterior density plots in Figure 2 of the main text and Supplementary Figures S5-S11.

References

Christen, J.A. and Fox, C. (2005) Markov chain Monte Carlo using an approximation. *Journal of Computational and Graphical statistics* **14**(4), 795-810.

Doucet, A. and Johansen, A. M. (2009) A tutorial on particle filtering and smoothing: Fifteen years later. *Handbook of nonlinear filtering* **12**(656-704), 3.

El Cheikh, R., Lepoutre, T., Bernard, S. (2012) Modeling biological rhythms in cell populations. *Mathematical Modelling of Natural Phenomena* **7**(6), 107-125.

Fearnhead, P., Giagos, V., Sherlock, C. (2014) Inference for reaction networks using the Linear Noise Approximation. *Biometrics* **70**(2), 457-466.

Golightly, A., Henderson, D.A., Sherlock, C. (2015) Delayed acceptance particle MCMC for exact inference in stochastic kinetic models. *Statistics and Computing* **25**(5), 1039-1055.

Goodwin, B.C. (1965) Oscillatory behavior in enzymatic control processes. *Advances in enzyme regulation* **3**, 425-437.

Heron, E.A., Finkenstädt, B., Rand, D.A. (2007) Bayesian inference for dynamic transcriptional regulation; the Hes1 system as a case study. *Bioinformatics* **23**(19), 2596-2603.

Roberts, G.O. and Rosenthal, J.S. (2009) Examples of Adaptive MCMC. *Journal of Computational and Graphical Statistics* **18**(2), 349-367.

Särkkä, S. (2007) On unscented Kalman filtering for state estimation of continuous-time nonlinear systems. *Automatic Control, IEEE Transactions on* **52**(9), 1631-1641.

Sherlock, C., Golightly, A., Henderson, D.A. (2017) Adaptive, delayed-acceptance MCMC for targets with expensive likelihoods. *Journal of Computational and Graphical Statistics* **26**(2), 434-444.

Singer, H. (2006) Continuous-discrete unscented Kalman filtering.

Smith, H. (2010) *An introduction to delay differential equations with applications to the life sciences* (Vol. 57). Springer Science & Business Media.

Suter, D.M. *et al.*, (2011) Mammalian genes are transcribed with widely different bursting kinetics. *Science* **332**(6028), 472-474.

Wilkinson, D.J. (2012) *Stochastic Modelling for Systems Biology.* Chapman & Hall.

Supplementary Figures and Tables

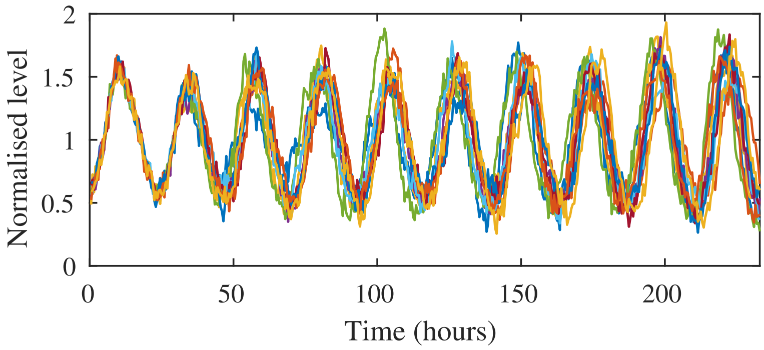

Figure S1. Molecule counts rescaled by their mean level, integrated over 0.5 hours and corrupted with measurement error. The assumed levels of signal to noise ratio is 20. SSA trajectories for the unobserved state are obtained according to reactions (8) and (9) in the main text, and assume parameters $R_{max} = 90$ molecules/h, $K_{pc} = 1.5 \times 10^2$ molecules, $n = 5$, $\mu = 0.25$ 1/h, $E[\tau] = 9.2$ h, $SD[\tau] = \sqrt{15}$ h, and $\tau_m = 30$ h. The initial condition is given by the first 30 hours of a sample *Cry1-luc* time series recorded in mice brain, aggregated de-trended and normalised.

	One step-ahead prediction	
	s/n=100	s/n=20
$\delta_t = 0.5$ h	0.96 (0.012)	0.94 (0.0096)
$\delta_t = 0.25$ h	0.97 (0.0091)	0.95 (0.012)
$\delta_t = 0.1$ h	0.96 (0.0068)	0.95 (0.014)
$\delta_t = 0.05$ h	0.96 (0.0052)	0.95 (0.015)
$\delta_t = 0.01$ h	0.95 (0.0053)	0.95 (0.016)
	Partial smoothing	
	s/n=100	s/n=20
$\delta_t = 0.5$ h	0.36 (0.036)	0.84 (0.044)
$\delta_t = 0.25$ h	0.81 (0.024)	0.93 (0.022)
$\delta_t = 0.1$ h	0.94 (0.0095)	0.95 (0.012)
$\delta_t = 0.05$ h	0.95 (0.0088)	0.95 (0.0088)
$\delta_t = 0.01$ h	0.95 (0.0086)	0.95 (0.0075)

Table S1. Empirical coverages (95% level) of the predictive and partial smoothing density provided by the filtering algorithm for the system under study. Values computed for two signal to noise ratios (s/n), 100 and 20, and for five discretisation time-intervals (δ_t). Mean (standard deviation) for 10 replicate simulated data-sets. Coverages are computed after the initial 30 hours accounting for the delay.

	Total	Prediction - covariance	Kalman update - covariance
$\delta_t = 0.5$ h	0.12 s (0.019)	0.02 s (0.002)	0.01 s (0.001)
$\delta_t = 0.25$ h	0.16 s (0.016)	0.04 s (0.004)	0.01 s (0.002)
$\delta_t = 0.1$ h	0.44 s (0.024)	0.22 s (0.012)	0.07 s (0.004)
$\delta_t = 0.05$ h	4.97 s (0.202)	3.73 s (0.146)	0.65 s (0.076)
$\delta_t = 0.01$ h	421.00 s (24.435)	393.04 s (22.490)	17.46 s (0.985)

Table S2. Running times (in seconds) of the filtering algorithm and selected sub-functions for the system under study. Values computed for five discretisation time-intervals (δ_t). Mean (standard deviation) for 10 replicate simulated data-sets. Simulations are run on a 1.7 GHz Intel Core i7 processor, with 8 GB of RAM.

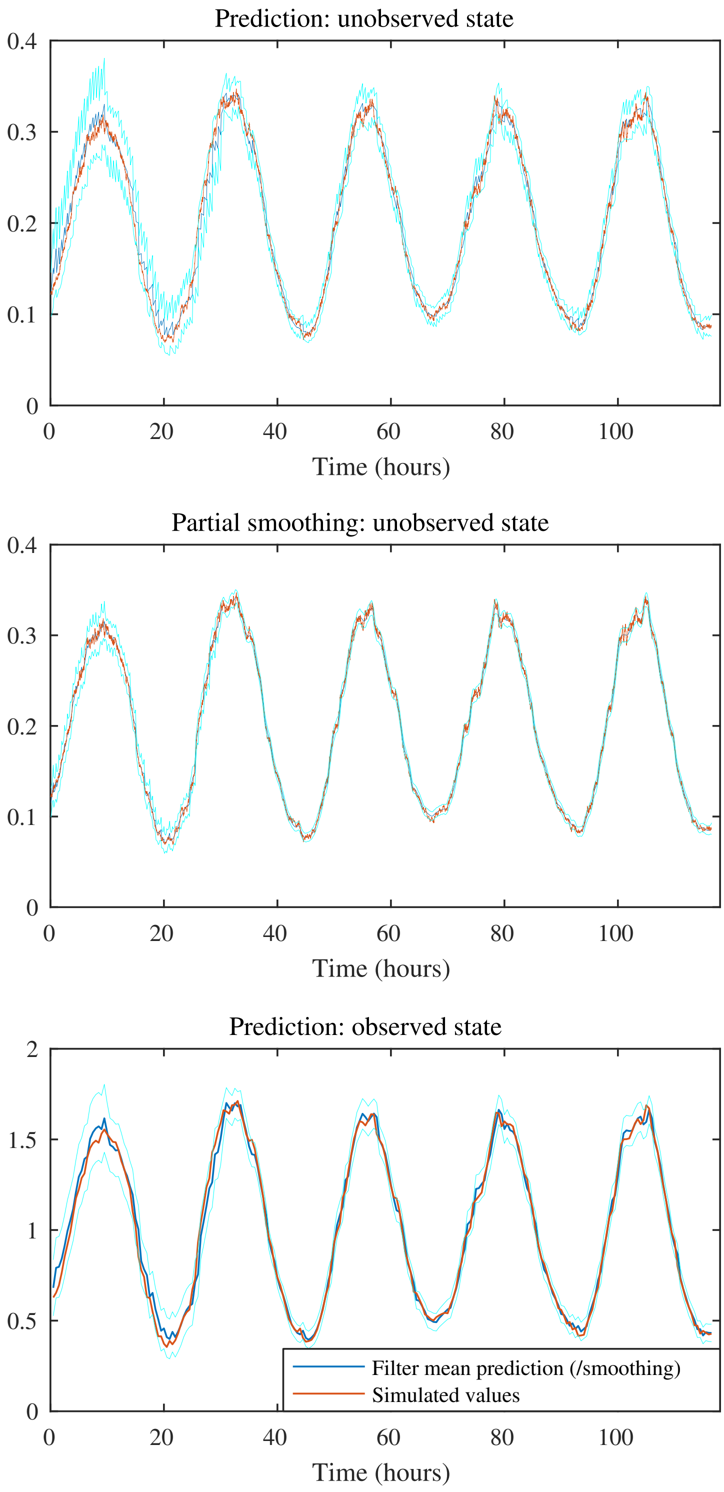

FIGURE S2. Unobserved state prediction (top), unobserved state partial smoothing (middle), and observed state prediction (bottom) for one sample simulated time-series of Figure 1 in the main text. The true simulated values are superimposed in red, the mean of the predictive or partial smoothing distribution is plotted in blue, while the light blue lines represent 95% variability intervals.

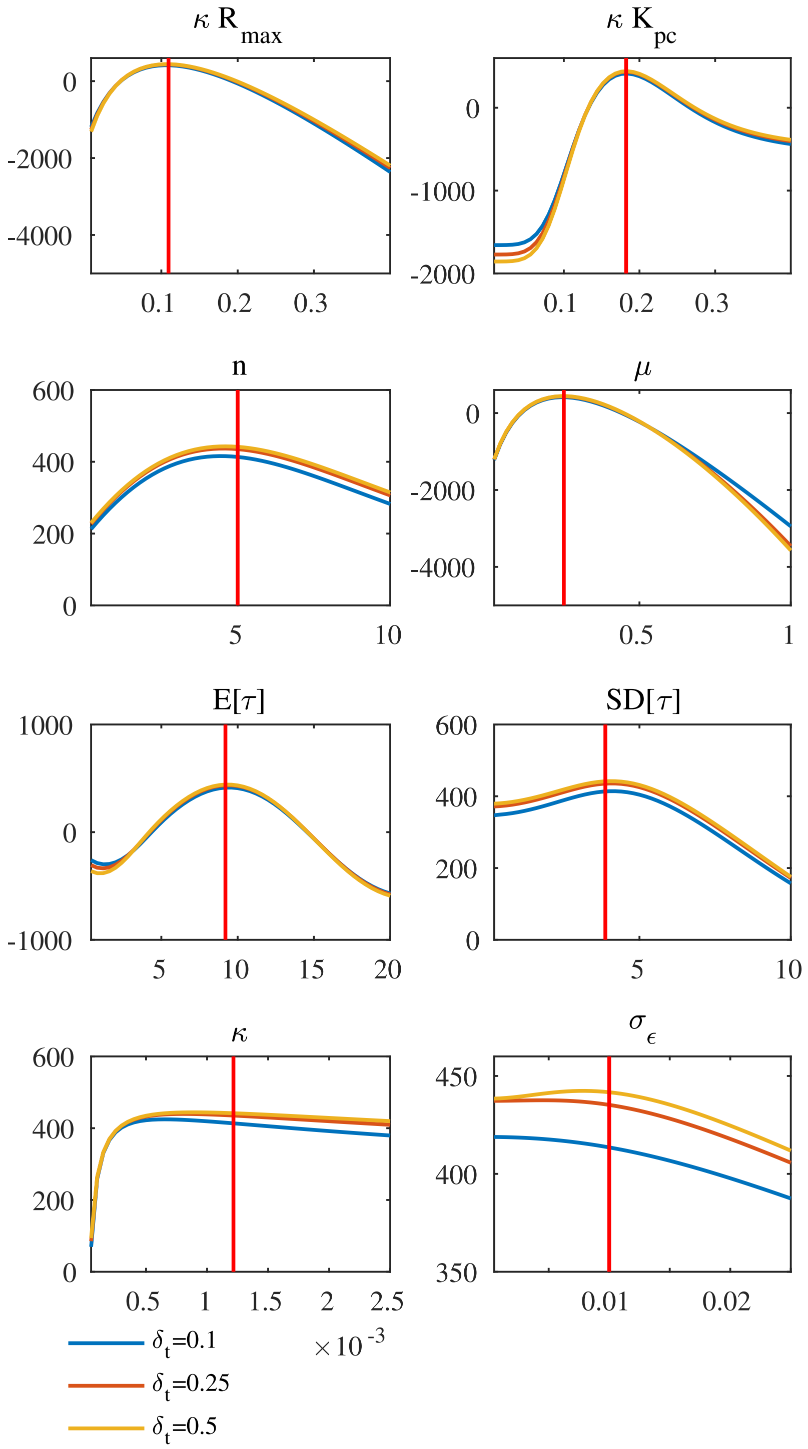

Figure S3. Univariate log-likelihood plots for the model parameters, excluding the parameters of the initial condition, for one sample simulated time-series of Figure 1 in the main text. The remaining parameters are set at their true simulation values (initial condition adjusted by visual inspection). The red line marks the true value (for $\delta_t = 0.1$). The signal to noise ratio is equal to 100.

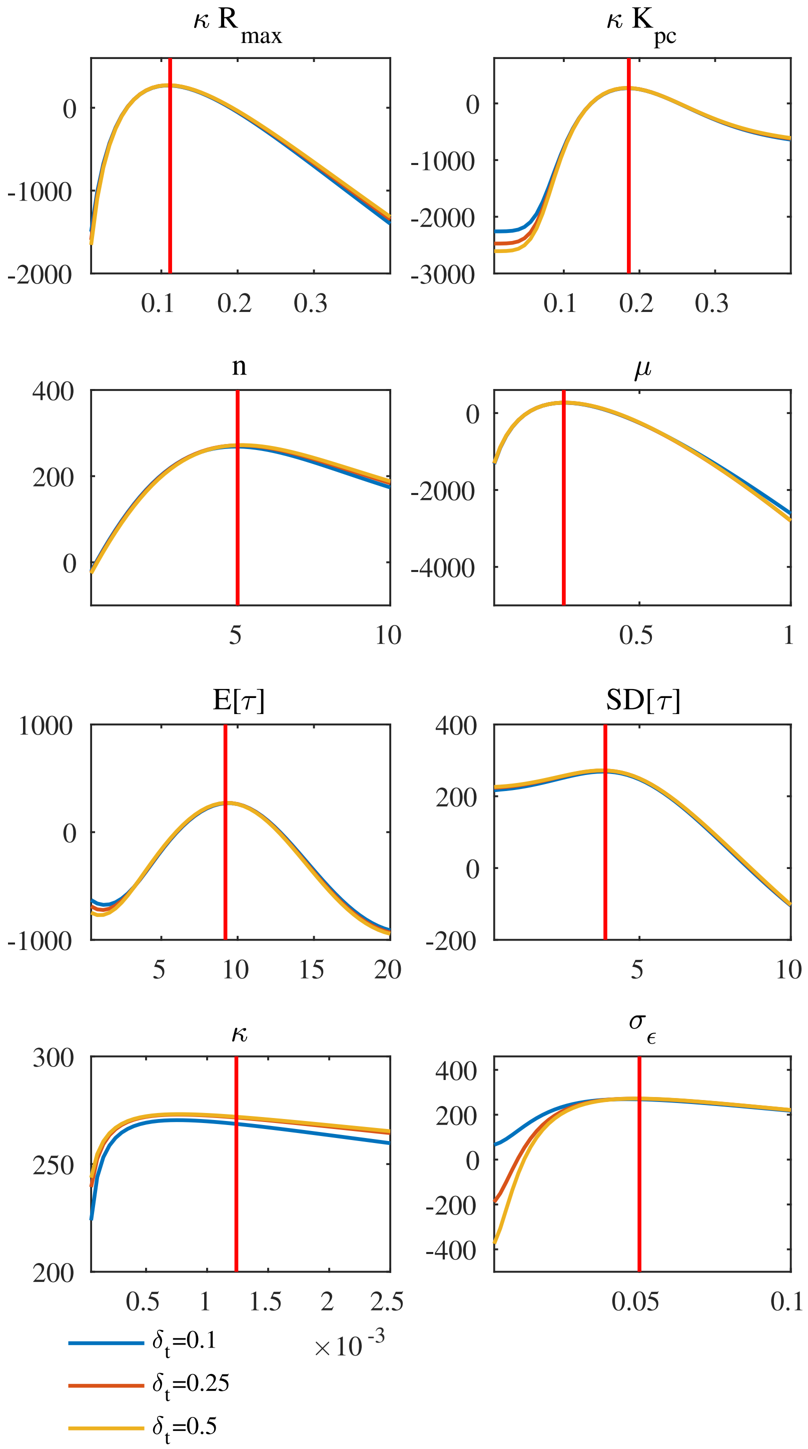

FIGURE S4. Univariate log-likelihood plots for the model parameters, excluding the parameters of the initial condition, for one sample simulated time-series of Figure S1. The remaining parameters are set at their true simulation values (initial condition adjusted by visual inspection). The red line marks the true value (for $\delta_t = 0.1$). The signal to noise ratio is equal to 20.

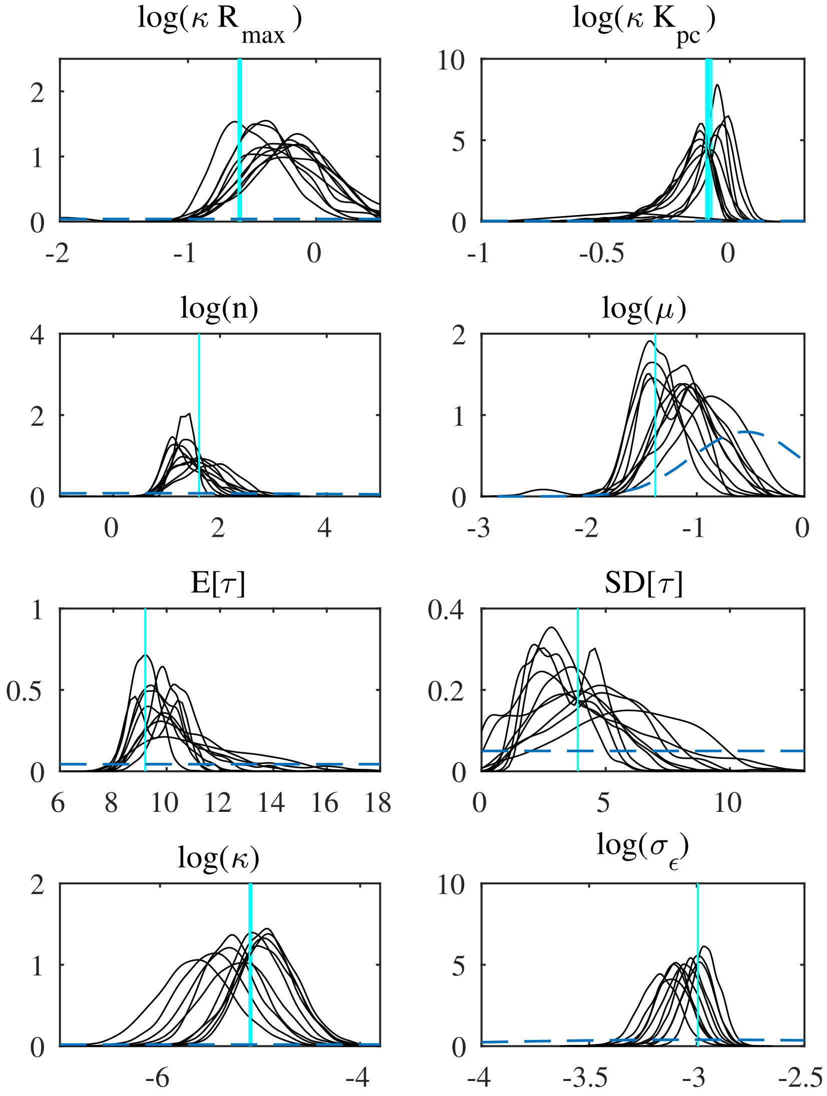

Figure S5. Kernel densities estimates of the model parameters posterior densities, excluding the parameters of the initial condition. The prior density is shown in red while the red vertical line marks the true value. Results for the simulated data shown in Figure S1, the signal to noise ratio is equal to 20 (one chain is excluded due to non-convergence).

	One step-ahead prediction	
	s/n=100	s/n=20
$\delta_t = 0.5$ h	0.85 (0.025)	0.87 (0.024)
$\delta_t = 0.25$ h	0.94 (0.022)	0.94 (0.025)
$\delta_t = 0.1$ h	0.96 (0.014)	0.95 (0.02)
$\delta_t = 0.05$ h	0.96 (0.014)	0.95 (0.019)
$\delta_t = 0.01$ h	0.95 (0.015)	0.95 (0.021)
	**Partial smoothing**	
	s/n=100	s/n=20
$\delta_t = 0.5$ h	0.28 (0.043)	0.74 (0.04)
$\delta_t = 0.25$ h	0.73 (0.026)	0.91 (0.019)
$\delta_t = 0.1$ h	0.92 (0.0092)	0.94 (0.015)
$\delta_t = 0.05$ h	0.95 (0.0075)	0.95 (0.014)
$\delta_t = 0.01$ h	0.95 (0.0061)	0.95 (0.014)

TABLE S3. Empirical coverages (95% level) of the predictive and partial smoothing density provided by the filtering algorithm for the system under study. Values computed for two signal to noise ratios (s/n), 100 and 20, and for five discretisation time-intervals (δ_t). Mean (standard deviation) for 10 replicate simulated data-sets. Coverages are computed after the initial 30 hours accounting for the delay. **Hill coefficient equal to 7**.

	One step-ahead prediction	
	s/n=100	s/n=20
$\delta_t = 0.5$ h	0.78 (0.027)	0.83 (0.043)
$\delta_t = 0.25$ h	0.93 (0.018)	0.93 (0.024)
$\delta_t = 0.1$ h	0.96 (0.016)	0.95 (0.02)
$\delta_t = 0.05$ h	0.96 (0.012)	0.95 (0.019)
$\delta_t = 0.01$ h	0.95 (0.012)	0.95 (0.018)
	Partial smoothing	
	s/n=100	s/n=20
$\delta_t = 0.5$ h	0.26 (0.019)	0.71 (0.036)
$\delta_t = 0.25$ h	0.68 (0.035)	0.9 (0.031)
$\delta_t = 0.1$ h	0.91 (0.017)	0.95 (0.022)
$\delta_t = 0.05$ h	0.94 (0.013)	0.95 (0.019)
$\delta_t = 0.01$ h	0.94 (0.01)	0.95 (0.017)

TABLE S4. Empirical coverages (95% level) of the predictive and partial smoothing density provided by the filtering algorithm for the system under study. Values computed for two signal to noise ratios (s/n), 100 and 20, and for five discretisation time-intervals (δ_t). Mean (standard deviation) for 10 replicate simulated data-sets. Coverages are computed after the initial 30 hours accounting for the delay. **Hill coefficient equal to 9**.

	One step-ahead prediction	
	s/n=100	s/n=20
$\delta_t = 0.5$ h	0.77 (0.027)	0.81 (0.031)
$\delta_t = 0.25$ h	0.92 (0.013)	0.92 (0.027)
$\delta_t = 0.1$ h	0.95 (0.0054)	0.95 (0.027)
$\delta_t = 0.05$ h	0.95 (0.0053)	0.95 (0.027)
$\delta_t = 0.01$ h	0.95 (0.0064)	0.95 (0.026)
	Partial smoothing	
	s/n=100	s/n=20
$\delta_t = 0.5$ h	0.26 (0.036)	0.68 (0.032)
$\delta_t = 0.25$ h	0.69 (0.021)	0.89 (0.025)
$\delta_t = 0.1$ h	0.92 (0.011)	0.95 (0.014)
$\delta_t = 0.05$ h	0.94 (0.0076)	0.96 (0.014)
$\delta_t = 0.01$ h	0.95 (0.0064)	0.96 (0.013)

TABLE S5. Empirical coverages (95% level) of the predictive and partial smoothing density provided by the filtering algorithm for the system under study. Values computed for two signal to noise ratios (s/n), 100 and 20, and for five discretisation time-intervals (δ_t). Mean (standard deviation) for 10 replicate simulated data-sets. Coverages are computed after the initial 30 hours accounting for the delay. **Hill coefficient equal to 11**.

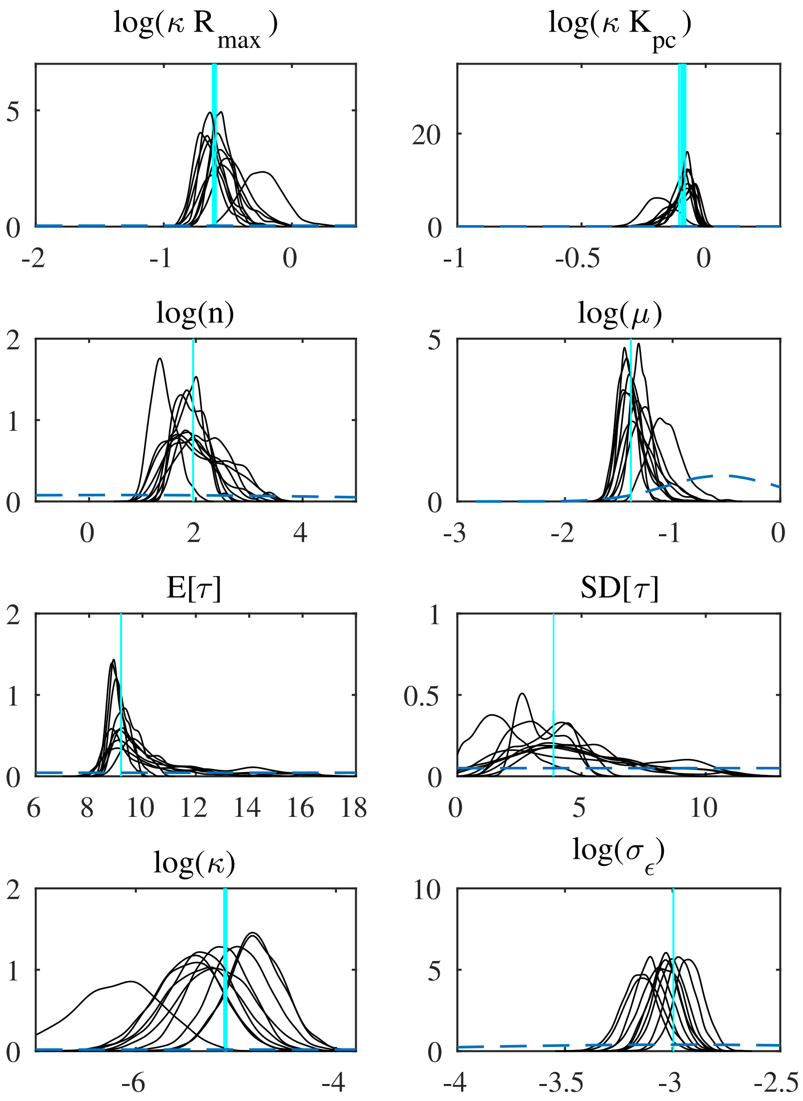

FIGURE S6. Kernel densities estimates of the model parameters posterior densities, excluding the parameters of the initial condition. The prior density is shown in red while the red vertical line marks the true value. The signal to noise ratio is equal to 20. **Hill coefficient equal to 7.**

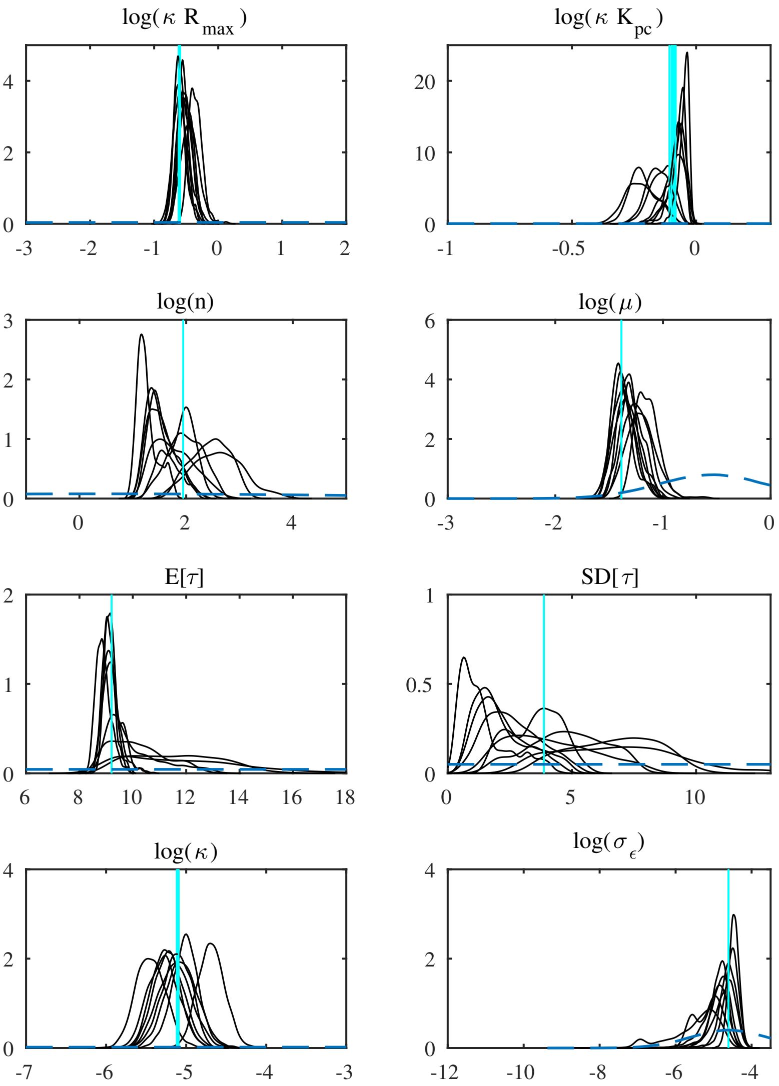

FIGURE S7. Kernel densities estimates of the model parameters posterior densities, excluding the parameters of the initial condition. The prior density is shown in red while the red vertical line marks the true value. The signal to noise ratio is equal to 100 (one chain is excluded due to non-convergence). **Hill coefficient equal to 7.**

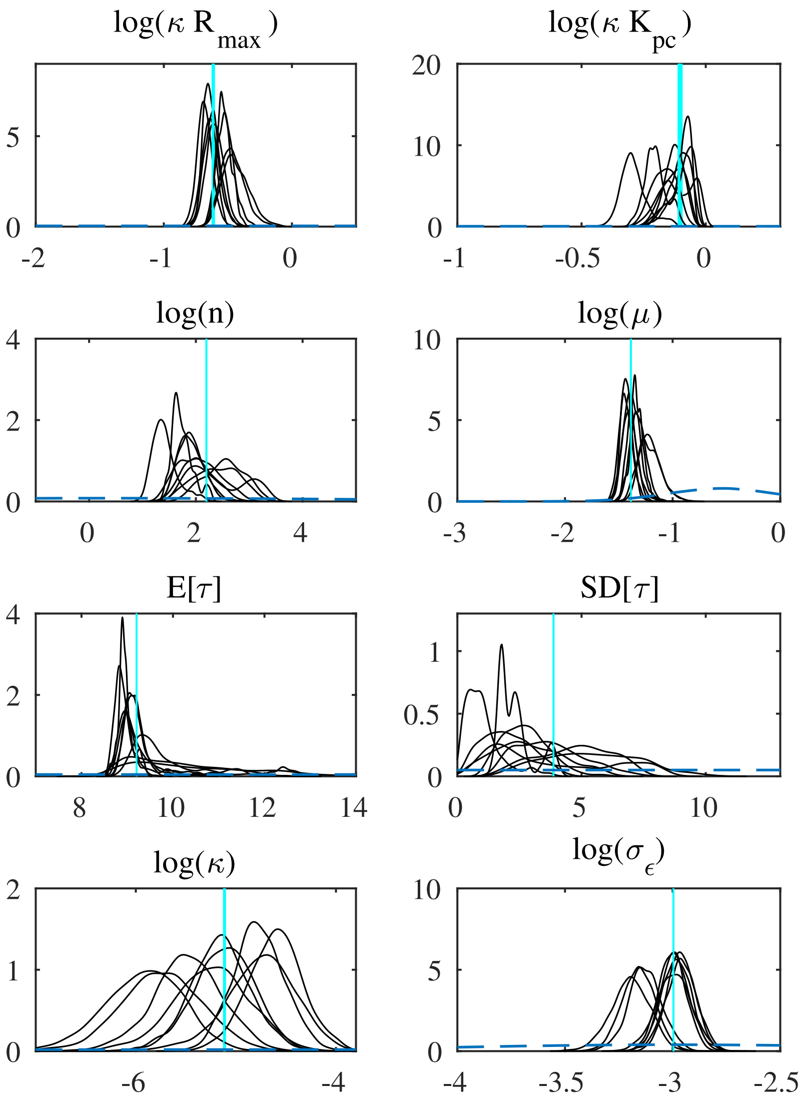

FIGURE S8. Kernel densities estimates of the model parameters posterior densities, excluding the parameters of the initial condition. The prior density is shown in red while the red vertical line marks the true value. The signal to noise ratio is equal to 20 (one chain is excluded due to non-convergence). **Hill coefficient equal to 9.**

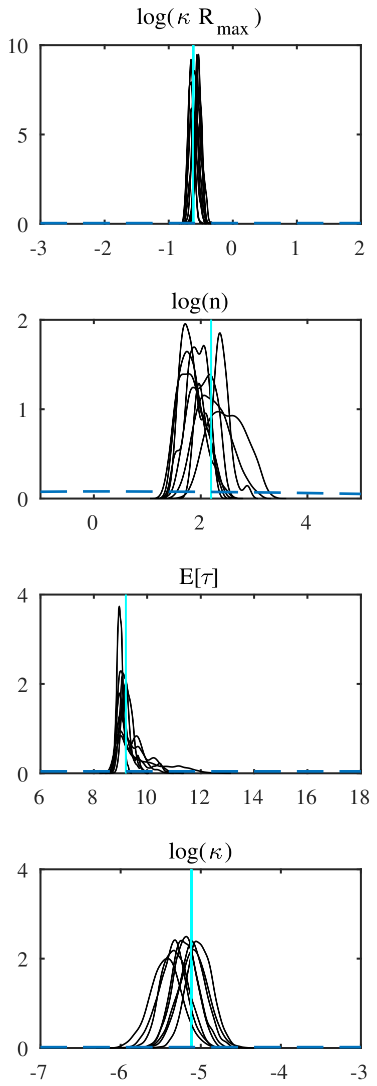

FIGURE S9. Kernel densities estimates of the model parameters posterior densities, excluding the parameters of the initial condition. The prior density is shown in red while the red vertical line marks the true value. The signal to noise ratio is equal to 100 (two chains are excluded due to non-convergence). **Hill coefficient equal to 9.**

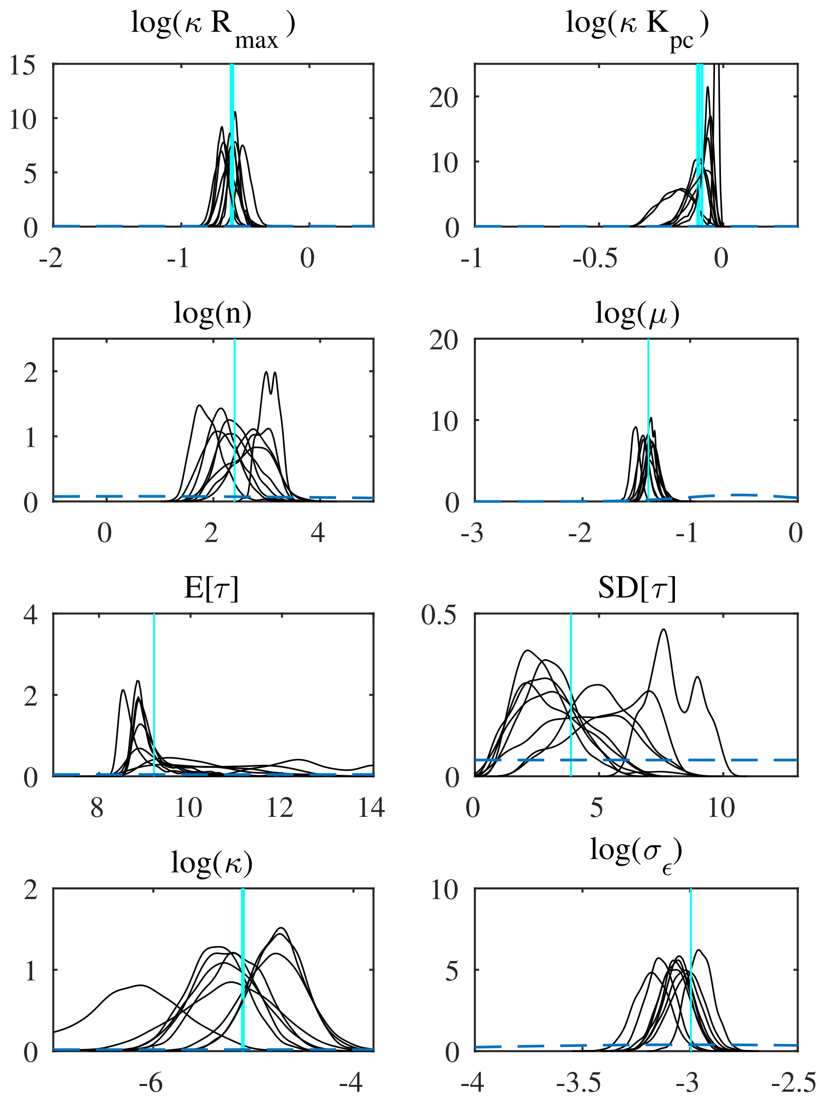

FIGURE S10. Kernel densities estimates of the model parameters posterior densities, excluding the parameters of the initial condition. The prior density is shown in red while the red vertical line marks the true value. The signal to noise ratio is equal to 20 (one chain is excluded due to non-convergence). **Hill coefficient equal to 11.**

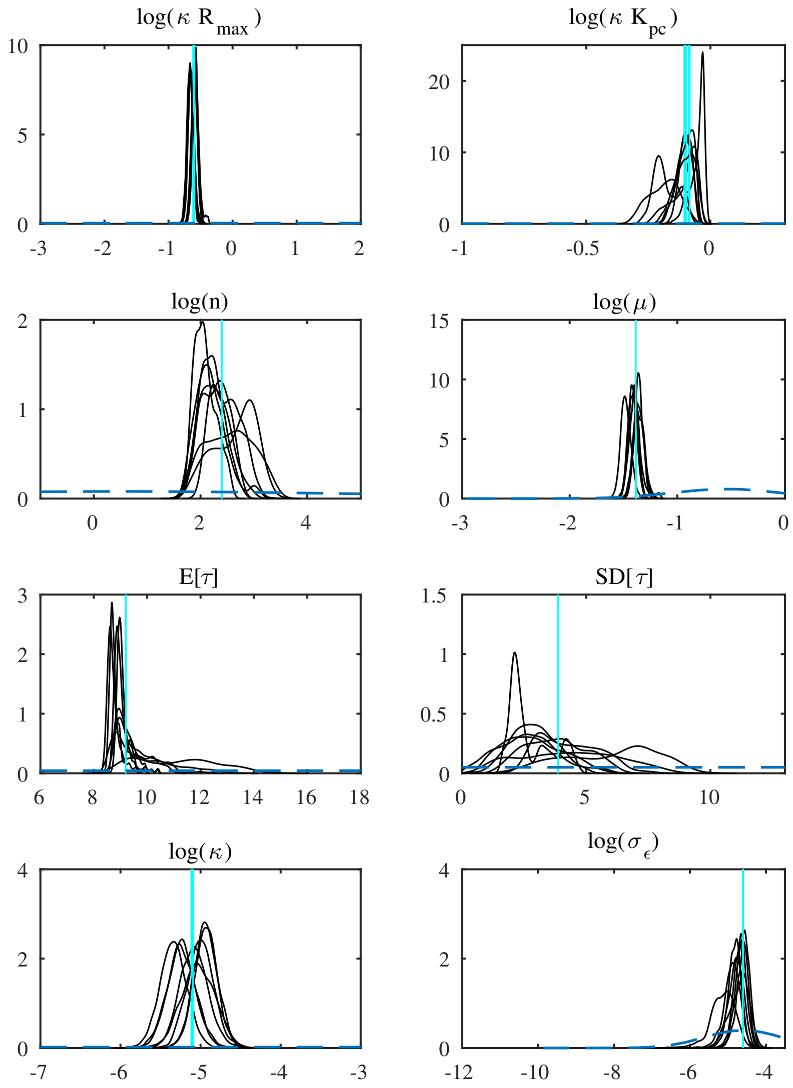

FIGURE S11. Kernel densities estimates of the model parameters posterior densities, excluding the parameters of the initial condition. The prior density is shown in red while the red vertical line marks the true value. The signal to noise ratio is equal to 100 (two chains are excluded due to non-convergence). **Hill coefficient equal to 11.**

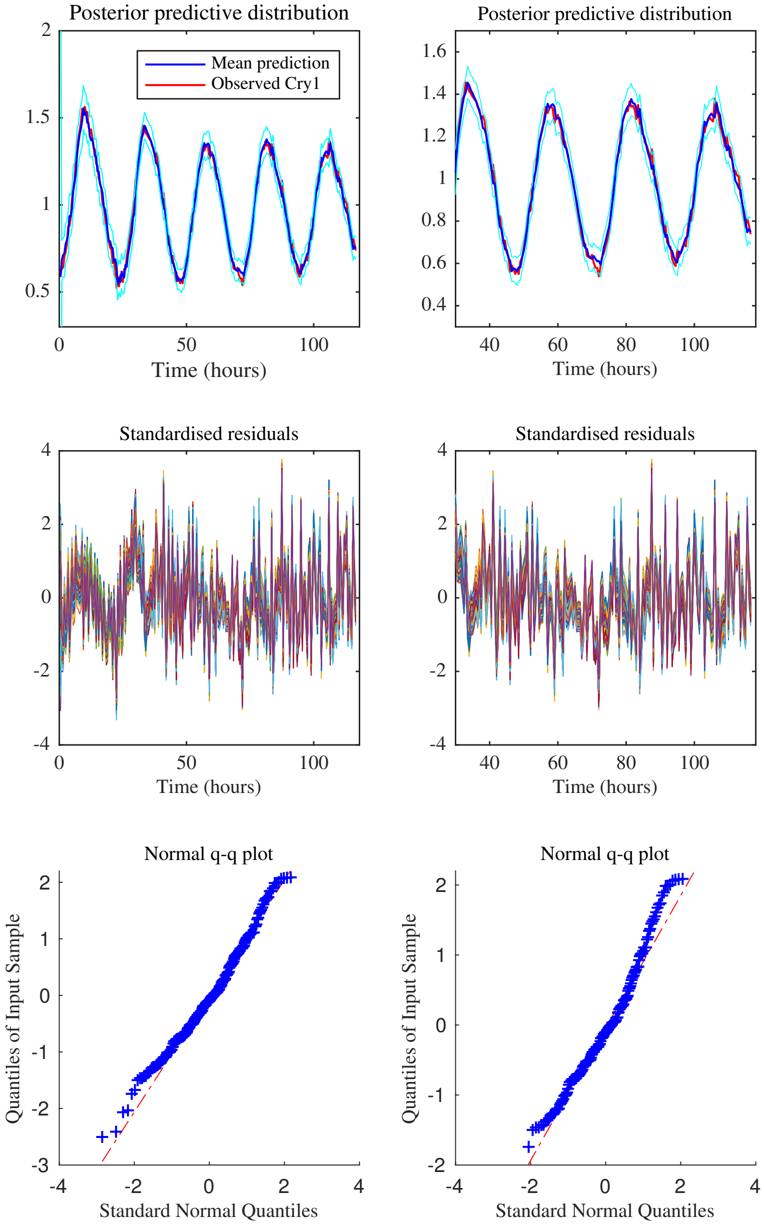

FIGURE S12. Model fit diagnostic plots for *Cry*1-*luc* data. Top: posterior predictive data density (median and 95% HPDI); Middle: standardised residuals; Bottom: *q*-*q* normal plot for the median standardised residuals. Samples are obtained from a thinned set of MCMC posterior parameter samples. Plots on the left concern the full observation time, while in the right plots the initial condition is discarded.
